# Immunoinformatics Approach Toward the Introduction of a Novel Multi-Epitope Vaccine Against *Clostridium difficile*

**DOI:** 10.3389/fimmu.2022.887061

**Published:** 2022-05-26

**Authors:** Caixia Tan, Fei Zhu, Yuanyuan Xiao, Yuqi Wu, Xiujuan Meng, Sidi Liu, Ting Liu, Siyao Chen, Juan Zhou, Chunhui Li, Anhua Wu

**Affiliations:** ^1^ Infection Control Center, Xiangya Hospital, Central South University, Changsha, China; ^2^ Center of Respiratory Medicine, Xiangya Hospital, Central South University, Changsha, China; ^3^ National Clinical Research Center for Geriatric Disorders (XiangYa Hospital), Changsha, China

**Keywords:** *Clostridium difficile*, multi-epitope vaccine, molecular docking, molecular dynamics simulation, immunoinformatics

## Abstract

*Clostridium difficile (C.difficile)* is an exclusively anaerobic, spore-forming, and Gram-positive pathogen that is the most common cause of nosocomial diarrhea and is becoming increasingly prevalent in the community. Because *C. difficile* is strictly anaerobic, spores that can survive for months in the external environment contribute to the persistence and diffusion of *C. difficile* within the healthcare environment and community. Antimicrobial therapy disrupts the natural intestinal flora, allowing spores to develop into propagules that colonize the colon and produce toxins, thus leading to antibiotic-associated diarrhea and pseudomembranous enteritis. However, there is no licensed vaccine to prevent *Clostridium difficile* infection (CDI). In this study, a multi-epitope vaccine was designed using modern computer methods. Two target proteins, CdeC, affecting spore germination, and fliD, affecting propagule colonization, were chosen to construct the vaccine so that it could simultaneously induce the immune response against two different forms (spore and propagule) of *C. difficile*. We obtained the protein sequences from the National Center for Biotechnology Information (NCBI) database. After the layers of filtration, 5 cytotoxic T-cell lymphocyte (CTL) epitopes, 5 helper T lymphocyte (HTL) epitopes, and 7 B-cell linear epitopes were finally selected for vaccine construction. Then, to enhance the immunogenicity of the designed vaccine, an adjuvant was added to construct the vaccine. The Prabi and RaptorX servers were used to predict the vaccine’s two- and three-dimensional (3D) structures, respectively. Additionally, we refined and validated the structures of the vaccine construct. Molecular docking and molecular dynamics (MD) simulation were performed to check the interaction model of the vaccine–Toll-like receptor (TLR) complexes, vaccine–major histocompatibility complex (MHC) complexes, and vaccine–B-cell receptor (BCR) complex. Furthermore, immune stimulation, population coverage, and *in silico* molecular cloning were also conducted. The foregoing findings suggest that the final formulated vaccine is promising against the pathogen, but more researchers are needed to verify it.

## 1 Introduction

*Clostridium difficile (C. difficile)* was isolated by Hall and O’Toole in 1935 and recently reclassified as *Clostridioides difficile* (1). In the late 1970s, Bartlett etal. (2) identified *C. difficile* as a pathogen related to antibiotic-associated diarrhea. *Clostridium difficile* infection (CDI) not only places a heavy burden on the healthcare system but also has a significant negative impact on the community population, causing nearly 230,000 CDIs in the United States each year, resulting in over 12,000 deaths and more than $1 billion in economic burden (3). The CDI epidemic occurs not only in the United States but also in Europe (4), Asia (5), and Australia (6). A significant issue with CDI is that it not only has a high incidence rate but also has a high recurrence rate. CDI occurs in approximately 15%–30% of patients treated for the first time and in about 45%–65% of patients previously diagnosed with recurrent *Clostridium difficile* infection (rCDI) (7). To better address the aforementioned issues, vaccination may be a viable option.

In recent years, several vaccines have been developed, and some are in phases of clinical testing, which can be roughly divided into two broad categories, toxin-based vaccines and nontoxic vaccines (8, 9). Currently, three toxin-based vaccines have entered phase II and above clinical studies. Both CDIFFENSE, a formalin-inactivated toxin vaccine, and VLE84, a recombinant toxin-based vaccine, have achieved satisfactory results in phase I and phase II trials (10, 11). Another formalin-inactivated toxin-based vaccine, developed by Pfizer, has completed phase III trials (12). However, Spencer etal. (13) found that toxin-based vaccines have no effect on *C. difficile* colonization or spore spreading from the host to the environment but may lead to an increased number of asymptomatic carriers (13, 14). Considering these limitations, several passive immunotherapies against CDI (15, 16) targeting other pathogenic factors, such as flagellum, S-layer proteins (SLPs), Cell wall protein 84 (Cwp84), CD0873, Pilin, and spore proteins, have been taken into consideration. However, some of these vaccine candidates failed to induce a sufficient level of immunity and provided only partial protection against CDI-related death (15, 17–19).

Immunoinformatics development has opened a new door for vaccine research. Compared with traditional vaccines, the multi-epitope vaccine has many advantages. For instance, they do not require microbial culture, saving time and cost; highly promiscuous epitopes can be recognized and combined by multiple alleles at a time to overcome the differences of alleles among the human population; biological harmfulness and toxicity reversion of traditional inactivated or attenuated vaccines can be avoided. At present, multi-epitope vaccines for different diseases, such as HIV-1 (20), cancer (21), *Chlamydia trachomatis* (22), and parasites (23), have been investigated.

Because *C. difficile* is strictly anaerobic, it can only survive outside the host intestine as a spore (24). Spores that are resistant to heat, dryness, and a plethora of commonly used disinfectants can transfer to disease-causing vegetative cells when exposed to an available environment (25). Thus, an ideal vaccine should target not only *C. difficile* propagules but also *C. difficile* spores.

The outermost layer of *C. difficile* spores plays a pivotal role in spore–host interactions and contributes to the persistence of the spore in the host (26). CdeC, a cysteine-rich spore exosporium protein, not only plays an essential role in exosporium morphogenesis and the correctness of spore coat assembly but also affects the physicochemical properties of the spore (27, 28). Studies have indicated that CdeC is an immunogenic protein that protects mice against *C. difficile* UK1 and *C. difficile* 630Δerm (29, 30).

The flagellum is equipped with multiple biological functions, contributing to biofilm formation, not merely playing an important role in pathogen motility but also participating in adhesion to host cells. Moreover, recent studies have shown that flagellum also affects the expression of toxin genes (31). Flagellum, as the ligand of Toll-like receptor (TLR)5 (31), can trigger the secretion of inflammatory cytokines through the TLR5 signaling pathway, thus playing the role of the immune modulator. Due to its unique immunocompetence, the flagellum has already been introduced as a favorable vaccine candidate, and some of these vaccines have already entered clinical trials (32). FliC (flagellin) and fliD (cap) proteins are two indispensable components of flagellum. FliD shows little variability from different *C. difficile* strains (33), unlike fliC, which has pronounced variability in the central domain that constitutes the exposed antigen part of the surface of the flagellum filament (34). According to a study, 87% of patients diagnosed with CDI were found to have fliD-specific antibodies, which was higher than the level of fliC-specific antibodies (21%) in Clostridium difficile associated diarrhea (CDAD) patients (35). Hence, in this study, CdeC and fliD were selected as target proteins to design a multi-epitope vaccine that attempted to defend against CDI by blocking spore and *C. difficile* propagant adherence to the host gut tract.

## 2 Methodology

### 2.1 Obtaining Target Protein Amino Acid Sequences

Not only the amino acid sequences of CdeC (CBE03081.1) and fliD (CBE01872.1) in Fasta format were retrieved from the protein database of the National Center for Biotechnology Information (NCBI) (https://www.ncbi.nlm.nih.gov/) but also the protein sequence of adjuvant LT-IIb (ID: 5G3L_H). The protein structure of TLR2 (ID: 2Z7X), TLR4 (ID:4G8A), TLR5 (ID:3J0A), B-cell receptor (BCR) (5DRW), major histocompatibility complex (MHC) I (ID:4U6Y), and MHC II (ID:5JLZ) molecules were downloaded from the Protein Data Bank using accession numbers (https://www.rcsb.org/).

### 2.2 Cytotoxic T-Cell Lymphocyte Epitope Prediction

The CTL epitopes were predicted by the NetCTL 1.2 server (http://www.cbs.dtu.dk/services/NetCTL/) that integrates the prediction of peptide MHC class I binding, proteasomal C terminal cleavage, and Transporter associated with Antigen processing (TAP) transport efficiency across 12 MHC class I supertypes (A1, A2, A3, A24, A26, B7, B8, B27, B39, B44, B58, and B62) in the human population (36). The antigenicity of predicted CTL epitopes was assessed using Vaxijen v2.0 (http://www.ddg-pharmfac.net/vaxijen/VaxiJen/VaxiJen.html) (37), and epitopes with an antigenicity greater than 0.4 were considered antigens (38). To enhance the antigenicity of the vaccine, only the epitopes with antigenicity greater than 1 were finally selected for the construction of the vaccine. Immunogenicity was further calculated by the Immune Epitope Database (IEDB) server (http://tools.iedb.org/immunogenicity/) (39). Additionally, the AllerTOP v. 2.0 (https://www.ddg-pharmfac.net/AllerTOP/index.html) (40) and ToxinPred (https://webs.iiitd.edu.in/raghava/toxinpred/index.html) (41) were employed to check the allergenicity and toxicity of epitopes, respectively. MHC class I allelic partners of the antigenic epitopes were predicted *via* Tepitool (http://tools.iedb.org/tepitool/) (42), and alleles with inhibitory concentration (IC50) values ≤500 nM and percentile rank scores ≤2 were considered in this study.

### 2.3 Helper T Lymphocyte Epitope Prediction

The NetMHC II pan 3.2 server (http://www.cbs.dtu.dk/services/2.5NetMHCIIpan/) (43) was utilized to predict epitopes with an affinity to MHC class II alleles. Based on the affinity for 14 human leukocyte antigen (HLA) DR alleles (HLA-DRB1*01:01; HLA-DRB1*03:01; HLA-DRB1*04:01; HLA-DRB1*04:04; HLA-DRB1*04:05; HLA-DRB1*07:01; HLA-DRB3*02:02; HLA-DRB1*09:01; HLA-DRB1*11:01; HLA-DRB1*13:02; HLA-DRB1*15:01; HLA-DRB3*01:01; HLA-DRB4*01:01; HLA-DRB5*01:01), all HTL epitopes were classified as three-level, and 2% and 10% were selected as the thresholds for the strong and weak binders, respectively. The HTL epitopes with a strong affinity to HLA DR alleles and inhibitory concentration (IC50) ≤500 nM were further tested for antigenicity. Similar to CTL epitopes, the allergenicity and toxicity of HTL epitopes were also predicted by the AllerTOP and ToxinPred servers, respectively. Interferon-gamma (IFN-γ) and interleukin-4 (IL-4) play a significant role in regulating immune cell development and differentiation as well as organism immune response (44, 45). Thus, antigenic (with a threshold value of >0.4), nonallergic, and nontoxic epitopes were tested to see if they could induce IFN-γ and IL-4 secretion using the INFepitope server (http://crdd.osdd.net/raghava/ifnepitope/) (46) and the IL-4pred server (https://webs.iiitd.edu.in/raghava/il4pre) (47), respectively. Only those IFN-γ- and IL-4-inducing epitopes were covered in the vaccine construct. In addition to IFN-γ and IL-4, there are still many cytokines involved in the development, maturation, activation, and effector processes of different immune cell subpopulations (48). Therefore, the ProInflam server (http://metagenomics.iiserb.ac.in/proinflam/index.html) (49), which predicts the pro-inflammatory response of peptides based on machine learning-based models, was used to predict the capacity of HTL epitopes to induce the secretion of pro-inflammatory cytokines like tumor necrosis factor (TNF), IL-1, IL-18, IL-12, or IL-23. IL-2, which was initially called T-cell growth factor, has been demonstrated to promote the proliferation of T cells and B cells and activate the cytotoxic function of natural killer (NK) cells, T lymphocytes, and monocytes (50). Therefore, we also have checked the capacity of selected HTL epitopes to induce the generation of IL-2 by the IL-2pred server (https://webs.iiitd.edu.in/raghava/il2pred/predict.php).

### 2.4 B-Cell Epitope Prediction

We used the ABCpred server (https://webs.iiitd.edu.in/raghava/abcpred/) (51) to predict linear B-cell epitopes using an artificial neural network, and a window length of 16 was selected as the predicted epitope length. Then, these selected epitopes were tested for antigenicity with VaxiJen v2.0, and epitopes with antigenicity equal to or greater than 0.9 were finally considered in this study. As before, AllerTOP v. 2.0 and ToxinPred were applied to predict the allergenicity and toxicity, respectively.

### 2.5 Prediction of Epitope Similarity With Human Proteins

To ensure that the final vaccine was incapable of inducing autoimmune disorders and cross-reactivity, all selected epitopes by the above steps were checked for their similarity to human proteins through the Blast (https://blast.ncbi.nlm.nih.gov/Blast.cgi) (52).

### 2.6 Multi-Epitope Vaccine Designation

The ultimately selected epitopes screened from CdeC and fliD in the previous steps were used for constructing the multi-epitope vaccine. The CTL and HTL epitopes were connected through KK linkers, while B-cell epitopes were linked together by GPGPG linkers. LT-IIb, a type II heat-labile enterotoxin (HLT), enables the formation of a greater proportion of effector memory cells by slowing CD8+ T-cell expansion and contraction dynamics (53). Thus, to improve the immunogenic capacity of the vaccine, LT-IIb was chosen as an adjuvant to attach to the N-terminus of the vaccine construct *via* the EAAAK linker.

### 2.7 Multi-Epitope Vaccine Features

#### 2.7.1 The Prediction of Physicochemical Properties of the Vaccine

We employed the ProtParam server (https://web.expasy.org/protparam/) (54) to predict the physical chemistry properties of the multi-epitope vaccine, including molecular weight, the number of amino acids, theoretical isoelectric point (pI), estimated half-life, instability index, aliphatic index, and grand average of hydropathicity (GRAVY). To comparatively evaluate the candidate vaccine’s properties, Brother of Regulator of Imprinted Sites (BORIS) antigens from both human and murine (C1) (55), a multi-epitope vaccine with immunodominant epitopes from six SARS-CoV-2 non-structural proteins and SARS-CoV-2 S protein (C2) (56), transmembrane protein 31 (TMEM31) (C3) (57), and a multi-epitope vaccine composed of immunodominant epitopes of (SYCP1) and ACRBP antigens (C4) (58) were chosen as positive controls.

#### 2.7.2 The Prediction of Antigenicity, Allergenicity, and Toxicity of the Vaccine

Antigenicity prediction of the final vaccine construct was performed using the Vaxijen v2.0, ANTIGENpro (http://scratch.proteomics.ics.uci.edu/), and Secret-AAR servers (http://microbiomics.ibt.unam.mx/tools/aar/) (59). According to cross-validation studies, the ANTIGENpro server generates protein antigenicity indices with a 76% accuracy using specific microarray data (60). The Secret-AAR servers evaluate the protein’s antigenic density and antigenic potential based on its Abundance of Antigenic Regions (AAR) value. And a lower AAR value implies better protein antigenicity. Additionally, both AlgPred (http://crdd.osdd.net/raghava/algpred/) and AllerTOP v2.0 were used to check the allergenicity of the vaccine, and the toxicity of the vaccine was predicted by the ToxinPred server.

#### 2.7.3 The Prediction of Transmembrane Domains, Probability of Solubility, and Signal Peptide of the Vaccine

We employed the TMHMM-2.0 to predict the transmembrane helices in the vaccine sequence. Based on multiple representations of the primary sequence, the SOLpro server (http://scratch.proteomics.ics.uci.edu/) predicted the propensity of a protein to be soluble upon overexpression in *E. coli* using a two-stage support vector machine (SVM) architecture with a precision of more than 74% (61). The signalP-6.0 online server (https://services.healthtech.dtu.dk/service.php?SignalP-6.0) (62) was employed to predict the signal peptide of the vaccine construct.

#### 2.7.4 The Prediction of Immunoglobulin A-Specific B-Cell Epitopes of the Vaccine

As is well-known, *C. difficile* is an intestinal pathogen, and intestinal mucosal immunity is very important for protection against CDI (63). Mucosal immunity is mainly mediated by secretory immunoglobulin A (s1gA) secreted by IgA plasma cells (64, 65). Thus, to clarify the potential of the vaccine to induce the generation of s1gA, the IgPred server (https://webs.iiitd.edu.in/raghava/igpred/index.html#) (66) was used to predict the presence of IgA-specific B-cell epitopes in the vaccine. The length of the epitope was set at 20 amino acid residues, the default length of the server, and the screening threshold of the epitope was set at 0.5. The server has a filter threshold of -1 to 1, with a higher threshold implying a high probability of correct prediction but poor coverage/sensitivity.

### 2.8 Secondary Structure Prediction

Since the protein secondary structure is a key indicator of protein folding, the Prabi server was employed (https://npsa-prabi.ibcp.fr/cgi-bin/npsa_automat.pl?page=/NPSA/npsa.gor4.html) to predict the secondary structure of the vaccine sequences based on the Garnier-Osguthorpe-Robson 4 (GOR4) method with an average accuracy of 64.4% (67).

### 2.9 Three-Dimensional Modeling, Refinement, and Validation

In this study, the 3D structure of the designed vaccine was predicted with the help of RaptorX (http://raptorx.uchicago.edu/), which was more suitable for the prediction of targets without close homologs in the protein structure, using an ultradeep convolutional residual neural network from a primary sequence or multiple sequence alignments (68). Then, the obtained models were refined using the GalaxyWEB server (http://galaxy.seoklab.org/), which could refine the non-conserved and less reliable zones of the protein’s 3D structure and further generate five refined models of each primary 3D model (69). Based on the model’s Global Distance Test-High Accuracy (GDT-HA), the root-mean-square deviation (RMSD), Molprobity, the best model was selected and further submitted to the PROCHECK, ERRAT (https://saves.mbi.ucla.edu/), and ProSA Web servers (https://prosa.services.came.sbg.ac.at/prosa.php) to verify its quality. The Ramachandran plot generated by PROCHECK reflects the stereochemical quality of a protein structure, and a superior model has more residues in the Ramachandran-favored region. ERRAT (70) assesses the protein structures by analyzing the statistics of non-bonded interactions between different atom types. A model with an ERRAT score of more than 85 is considered to be of high quality, and a higher score indicates a better model. The Z score generated by the ProSA Web server evaluates the overall quality of the protein’s 3D structures, and a positive value indicates that there are errors in the 3D structures of the protein (71). 

### 2.10 Molecular Docking

MHC molecules, which are involved in the recognition and presentation of antigenic peptides, are important for the activation of CD8+ T cells and CD4+ T cells (72). TLRs recognize pathogen-associated molecular patterns (PAMPs) on microorganisms when interacting with organisms, thus activating innate immune cells and inducing the expression and the secretion of a variety of pro-inflammatory cytokines. TLR4 and TLR2, which are expressed on the surface of a subgroup of immunological cells, are capable of recognizing a wide range of bacterial surface proteins, such as lipoteichoic acid (LTA), peptidoglycan (PGN), and lipoarabinomannan (73, 74). TLR5 is known to recognize flagellin proteins (75). B cells are responsible for the body’s humoral immune response (76). BCR acts as a gatekeeper on the surface of B cells; only those epitopes recognized and bound by BCR can further activate B cells. Therefore, predicting the interactions between the designed vaccine and TLRs (TLR2, TLR4, and TLR5), MHC (HLA-A*02:01 and HLA-DRB1*0401) molecules and BCR were strongly necessary. In this study, we first used the HADDOCK 2.4 server (https://wenmr.science.uu.nl/haddock2.4/) (77) to perform the molecular docking between vaccine and TLRs and MHC molecules. The clusPro server (78) has an antibody mode that provides a receptor mask of the non-complementarity determining regions (non-CDRs) that can be precisely complementary to the antigenic determinant of the protein, which indicated that the clusPro was more suitable to perform the molecular docking between vaccine and BCR. Thus, clusPro (https://cluspro.org/tutantibody.php) was selected to conduct the molecular docking for vaccine and BCR in this study. Meanwhile, to further verify vaccine affinity between vaccine and receptors, the docking between vaccine and receptors (TLRs, MHC molecules, and BCR) was also performed by Rosetta software (79). The TLRs and MHC molecules act as receptors, and the designed vaccine acts as a ligand. The detailed docking steps were as follows: (1) A box with the greatest range around the receptor’s geometric center was formed, and amino acids within 1.2 nm of the binding region on the vaccine were placed into the grid box for docking. The different types of atoms were then used as probes to scan and calculate the grid energy; (2) Rosetta software performed a conformational search for the ligand within the range of the box, and the final rankings were determined according to the scores based on the conformation, orientation, position, and energy of the ligand; (3) A total of 1,000 intermolecular interaction phases were obtained, and the ranking was performed based on the scores of molecular docking energy; (4) Cluster analysis was conducted by the built-in cluster analysis module of Rosetta software, and the complex system with the maximum clustering phase and the lowest binding energy score was chosen for further research.

### 2.11 Molecular Dynamics Simulation

Molecular dynamics (MD) simulation was conducted by Amber18 software (80, 81) to better understand the dynamics and stability of the optional vaccine–receptor docking complexes, and it runs a total of 80 ns. Initially, the Amber99SB force field (82) was applied to generate the parameters of vaccine and receptors, and the force field of heavy atoms with different bond angles was set. Then, taking the protein complex system as the center, adding a cubic water box of 1 nm and Na+ to make the system electrically neutral and preserve the topology and coordinate structure stable. During the preprocessing stage, two-step energy minimization was performed, first restricting the protein to minimize the energy of the water molecule and then releasing the protein to minimize the energy of the whole system. The first energy minimization was performed over 5,000 cycles, with 1,500 cycles using the fastest descent method; the second energy minimization was run over 5,000 cycles, with 2,000 cycles using the fastest descent method. The system temperature gradually increased from 0 to 300 K, and the system pressure was equilibrated under the protocol. The Langevin temperature control method and Berendsen pressure control method were employed to balance the temperature and pressure for 100 ps, respectively. The MD simulation was performed for 80 ns. The cutoff distance between van der Waals energy and short-range electrostatic energy was set at 10 Å, and the long-range electrostatic energy was calculated employing the particle mesh Ewald (PME) method during the run time. Moreover, the RMSD value of the complex system were monitored in real time until the expected average structure of the protein complexes was obtained for subsequent binding mode analysis.

### 2.12 Population Coverage

HLA alleles are extremely polymorphic, and different HLA types are expressed at significantly different frequencies in different races. Since the selected epitope will induce an immune response only in individuals expressing HLA alleles that can bind to the specific epitope, we used the IEDB tool (http://tools.iedb.org/population/) to predict the population coverage of the designed vaccine in different areas with an algorithm based on HLA allele binding and HLA allele frequencies (83).

### 2.13 Immune Simulation

To clarify whether the designed vaccine can induce the protective immune response of the host, we employed the C-ImmSim server (https://kraken.iac.rm.cnr.it/CIMMSIM/index.php?page=0) (84) that utilizes position-specific scoring matrices (PSSMs) to describe the immune response between the host and antigen to conduct an *in silico* immune simulation. The detailed immunization regimen included three injections of 1,000 vaccine units each, with a 4-week interval between each injection. In this investigation, the whole immune simulation process ran for 1,050 time steps, and each time step represents 8 h, with the injection points set at time steps 1, 84, and 168 in this study. HLA-A*02:01, HLA-B*07:02, and HLA-DRB1*0101 were selected as host MHC molecular combinations, and other parameters remained at the default.

### 2.14* In Silico* Molecular Cloning

**Figure 1:**
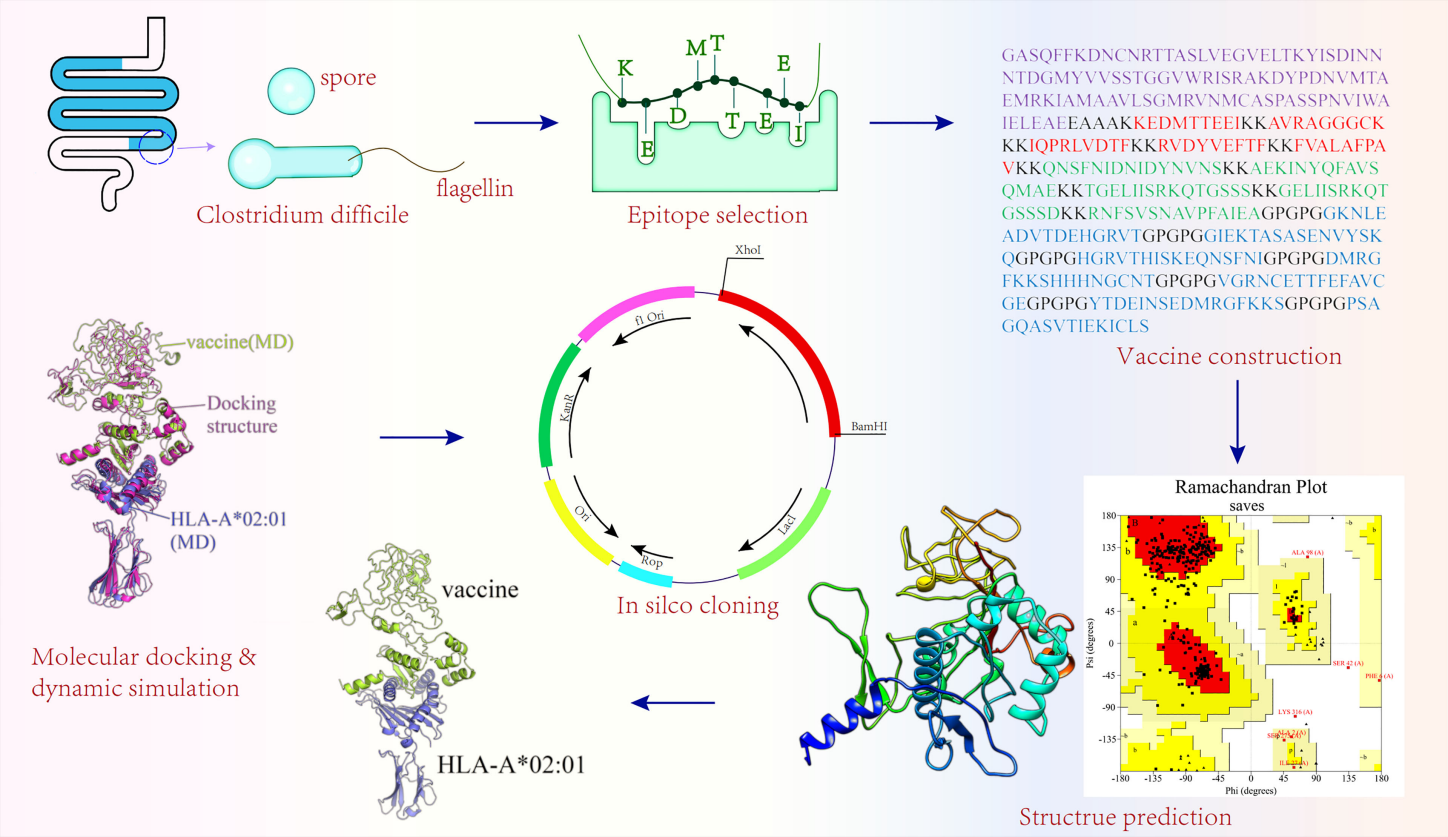
Flowchart of the multi-epitope vaccine construction.

It is well known that *E. coli* is the most commonly used prokaryotic expression system (85). *E. coli* (K12 strains) was selected as the host organism in this study. To achieve better expression of the vaccine in *E. coli*, the JCAT online server (http://jcat.de/) (86) performed reverse translation and codon optimization of the vaccine. Two parameters of the JCAT server, codon adaptive index (CAI) and the GC content, were used to evaluate the expression potential of vaccine in host. The CAI refers to the consistency between the use frequency of synonymous codons and the optimal codon. The CAI value ranges from 0 to 1, and the closer it is to 1, the better the consistency between the codon and the optimal codon. The GC content refers to the ratio of guanine (G) and cytosine (C) in the nucleotide sequence, and its value range should be between 30% and 70%; if not, the transcription and translation efficiencies will be greatly discounted. XhoI and BamHI restriction sites were added to the N- and C-terminal sites of optimized codon sequences, respectively. The pET28a(+) plasmid, one of the most popular expression plasmids on the market (described in >40,000 published articles) (87), is good for cloning and subcloning heterologous proteins with the *E. coli* as the host (21, 88). The pET28a(+) plasmid enables high titers of recombinant protein production—as much as 50% of the total expression product after a few hours of induction because of containing T7 promoter (89). Meanwhile, the pET-28a(+) can easily weaken the protein expression by reducing the concentration of inducers [Isopropyl â-D-1-thiogalactopyranoside (IPTG)]. Under non-inducible conditions, the target gene can be silenced completely without transcription (89). Additionally, pET-28a(+) not only provides different tags (N-terminal: His/Thrombin/T7 protein tag and C-terminal: His tag) but also has appropriate restriction sites, which were beneficial to the purification of the target protein and cloning of different genes (90). However, Shilling etal. (87) discovered two design flaws in pET vectors. One is the lack of a complete T7 consensus sequence (T7pCONS) of the T7lac promoter in the traditional pET28a(+) vector, and the other is that the compatibility of the TIR, recognized by the 30S ribosomal subunit during translation initiation, in the traditional pET28a(+) vector with the ribosome of *E. coli*, is not at its best (87); both defects would impact the production efficiency of the protein. Furthermore, leaky expression may occur in some hosts. There may still be some low-level expression of T7 RNA polymerase from the LacUV5 promoter in some expression host strains even if IPTG is not used (89), which may result in some genes, harmful to target bacteria, expressed in some host strains. Finally, the vaccination sequence was inserted into pET28a(+) expression vectors with the help of SnapGene in this study. The flowchart of the multi-epitope vaccine construction is shown in **Figure 1**.

## 3 Results

### 3.1 Prediction and Screening of Epitopes

CTLs, HTLs, and B cells are important participants in the human protective immune response. CTLs indirectly orchestrate human adaptive immune responses by secreting and aggregating a variety of cytokines, in addition to directly mediating the death of target cells (91). HTLs not only can activate T cells and B cells by recognizing presented foreign antigens and secreting certain cytokines but also contribute to B-cell differentiation into memory B cells (92). B cells are important for the generation of humoral immunity and long-lasting immunity (93). Hence, an ideal vaccine should include both T-cell epitopes (CTL and HTL epitopes) and B-cell epitopes. After layers of filtration, 5 CTL epitopes (**Table 1**), 5 HTL epitopes (**Table 2**), and 7 linear B-cell epitopes (**Table 3**) were finally selected for vaccine construction, and detailed screening materials are provided in the supplementary materials (**Tables S1–S3**). Among the 5 final selected HTL epitopes, which were all IFN-γ-inducing and IL-4-inducing epitopes, 3 of 5 HTL epitopes were predicted to be inducing pro-inflammatory cytokines. Similarly, 3 of 5 selected HTL epitopes were predicted as IL-2 inducing. From these, we can know that relatively few HTL epitopes among our selected ones are responsible for inducing multiple immune factors, but the combination of multiple epitopes would induce a strong immune response in the body.

**Table 1:** Final selected cytotoxic T lymphocyte (CTL) epitopes for vaccine construction.

Protein	Start position	Epitope	Combined score	Antigenicity	Immunogenicity	Toxicity	Allergenicity	IC50	Rank%	Best binding allele
CdeC	267	FVALAFPAV	1.2506	1.0868	0.16334	–	–	3.04	0.02	HLA-A*02:06
								7.99	0.06	HLA-A*68:02
								11.86	0.11	HLA-A*02:01
								44.18	0.1	HLA-C*12:03
								48.1	0.12	HLA-C*03:03
								48.1	0.12	HLA-C*03:04
								56.61	0.21	HLA-C*03:02
								116.79	0.35	HLA-C*16:01
								144.08	0.11	HLA-C*12:02
								414.92	0.24	HLA-C*17:01
	274	AVRAGGGCK	0.8519	2.1514	0.11817	–	–	22.67	0.09	HLA-A*30:01
								235.49	0.58	HLA-A*03:01
	284	RVDYVEFTF	0.8507	1.2835	0.25941	–	–	30.86	0.04	HLA-A*32:01
								97.76	0.34	HLA-B*58:01
								107.78	0.09	HLA-C*05:01
								301.46	0.13	HLA-C*08:02
								327.37	1.5	HLA-B*15:25
								391.92	0.15	HLA-B*13:01
	375	IQPRLVDTF	1.4753	1.3873	0.11794	–	–	297.47	0.31	HLA-A*24:02
								315.09	0.45	HLA-A*23:01
								406.99	1.1	HLA-B*15:01
FliD	321	KEDMTTEEI	0.9598	1.2159	0.04784	–	–	108.87	0.18	HLA-B*40:01

Final selected CTL epitopes. The start position represents the starting site of the first amino acid residue of the epitope. The default minimum threshold value of the combined score was set at 0.75. Epitopes with antigenicity score >0.4 are considered to be antigenic, which can specifically bind to the antibodies or sensitized lymphocytes. Epitopes with immunogenicity score >0 are considered to be immunogenic, which can elicit an immune response. In addition, epitopes with the half-maximal inhibitory concentration (IC50) ≤500 nm and Rank% ≤2% are considered to have good binding ability to human leukocyte antigen (HLA) I class alleles. “-” refers to negative, and “+” refers to positive.

**Table 2:** Final selected Helper T lymphocyte (HTL) epitopes for vaccine construction.

Protein	Start position	Epitope	Best binding allelle	IC50	Rank%	Antigenicity	Allergenicity	Toxicity	INF-γ	IL-4	IL-2	Pro-C
CdeC	120	RNFSVSNAVPFAIEA	HLA-DRB1*0701	5.99	0.35	0.4815	-	-	+	+	+	+
			HLA-DRB1*0901	11.96	0.22							
FliD	254	QNSFNIDNIDYNVNS	HLA-DRB3*0101	147.38	1.11	1.0266	-	-	+	+	+	+
			HLA-DRB1*0401	365.28	1.39							
	100	AEKINYQFAVSQMAE	HLA-DRB1*0103	218.58	0.93	0.8263	-	-	+	+	+	+
			HLA-DRB1*0701	38.8	0.86							
			HLA-DRB4*0101	181.32	1.82							
			HLA-DRB1*0101	6.42	0.63							
			HLA-DRB1*0401	33.58	1.03							
			HLA-DRB1*0901	30.48	0.21							
	181	TGELIISRKQTGSSS	HLA-DRB1*1101	67.5	0.3	0.4792	-	-	+	+	-	-
	182	GELIISRKQTGSSSD	HLA-DRB1*1101	79.46	0.8	0.4725	-	-	+	+	-	-

HTL epitopes predicted using NetMHC II pan 3.2 server. The start position represents the starting site of the first amino acid residue of the epitope. Epitopes with antigenicity score >0.4 are considered to be antigenic, which can specifically bind to the antibodies or sensitized lymphocytes. Epitopes with immunogenicity score >0 are considered to be immunogenic, which can elicit an immune response. In addition, epitopes with the half-maximal inhibitory concentration (IC50) ≤500 nm and Rank% ≤2% are considered to have good binding ability to human leukocyte antigen (HLA)II class alleles. Pro-C refers to whether the HTL epitope can induce the secretion of pro-inflammatory cytokines like TNF, IL-1, IL-18, IL-12, or IL-23. "-" refers to negative, "+" refers to positive.

**Table 3:** Final B-cell epitopes selected for vaccine construction.

Protein	Start position	Epitope	Score	Antigenicity	Allergenicity	Toxicity
FliD	244	HGRVTHISKEQNSFNI	0.79	0.9436	-	-
	443	GIEKTASASENVYSKQ	0.77	0.9803	-	-
	233	GKNLEADVTDEHGRVT	0.72	1.5221	-	-
CdeC	33	DMRGFKKSHHHNGCNT	0.89	1.0467	-	-
	211	VGRNCETTFEFAVCGE	0.86	1.4242	-	-
	25	YTDEINSEDMRGFKKS	0.8	0.9188	-	-
	171	PSAGQASVTIEKICLS	0.8	0.975	-	-

B-cell epitopes predicted using ABCpred server. The start position represents the starting site of the first amino acid residue of the epitope. The default minimum threshold value of the score was set at 0.51. Epitopes with antigenicity score>0.4 are considered to be antigenic, which can specifically bind to the antibodies or sensitized lymphocytes "-" refers to negative.

### 3.2 The Multi-Epitope Vaccine Features

#### 3.2.1 The Prediction of Physicochemical Properties of the Vaccine

**Figure 2:**
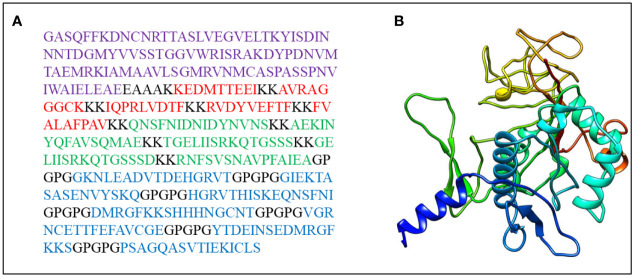
**(A)** The final vaccine sequence; purple denotes adjuvant, Cytotoxicity T Lymphocytes (CTL) epitopes are colored red, Helper T lymphocyte (HTL) epitopes are colored green, B-cell epitopes are colored blue, and all of them are coupled *via* different connectors. **(B)** The three dimensional (3D) structure of the vaccine.

The final vaccine construct (**Figure 2A**) is composed of four parts, CTL epitopes, HTL epitopes, linear B-cell epitopes, and LT-IIb (adjuvant), containing 389 amino acids, and its molecular weight was 41,632.89 Da. The vaccine pI is 9.11, and its negatively charged residues and positively charged residues are 43 and 53, respectively. The designed vaccine was considered stable and thermostable with an instability index II of 31.98 and an aliphatic index score of 59.43. Generally, a protein with an instability index below 40 was stable. Additionally, the higher aliphatic index of the protein results in better thermal stability (94). The GRAVY score was -0.585, and a negative sign indicated that it was hydrophilic (95). The estimated half-life of the designed vaccine is 30 h in mammalian reticulocytes, greater than 20 h in yeast, and greater than 10 h in *E. coli*. The physicochemical parameters of the final vaccine construct were compared to those of positive controls (**Table 4**). Compared with C1, C2, and C3, the aliphatic index of the final vaccine construct is relatively small, but it is better than C4 and some other multi-epitope vaccines (96–98). Overall, the final vaccine construct revealed appropriate physicochemical properties, including well solubility and stability, which contributed to enhancing the vaccine’s bioavailability and immunogenicity and lessening side effects.

**Table 4:** Comparison of several physicochemical properties of the positive vaccine controls and *C. difficile* candidate vaccine.

Properties	C1	C2	C3	C4	Vaccine candidate
Molecular weight	47 kDa	51.64 kDa	39.83 kDa	6.38 kDa	41.63 kDa
Isoelectric point	9.35	10	9.61	9.61	9.11
Instability index	28.05	27.09	30.03	33.38	31.98
Aliphatic index	67.45	79	84.57	53.53	59.43
GRAVY	-0.452	-0.354	-2.15	-1.127	-5.585
Antigenicity^a^	0.5286	0.59	—	0.61	0.97
Antigenicity^b^	—	0.74	—	0.62	0.94
Antigenicity^c^	—	39.8	—	35.4	30

Antigenicity^a^, Antigenicity predicted by Vaxijen 2.0; Antigenicity^b^, Antigenicity predicted by ANTIGENpro; Antigenicity^c^, Antigenicity predicted by Secret-AAR servers. GRAVY refers to grand average of hydropathicity.

#### 3.2.2 The Prediction of Antigenicity, Allergenicity, and Toxicity of the Vaccine

The antigenicity scores of the designed vaccine were 0.97, 0.94, and 30 as predicted by the Vaxijen 2.0, ANTIGENpro, and Secret-AAR servers, respectively, showing that the vaccine has a strong affinity to the host immune system products (**Table 4**). And the vaccine was non-allergenic and nontoxic.

#### 3.2.3 The Prediction of Transmembrane Domains, Solubility, and Signal Peptide of the Vaccine

The designed vaccine detected no transmembrane helix (**Figure S1**), The SQLpro server predicted that the probability of solubility of the multi-epitope vaccine upon overexpression in *E. coli* was 0.61 and the prediction results of the TMHMM-2.0 server showed that the vaccine was an extracellular protein, which indicated that the multi-epitope vaccine protein could be soluble expressed and secreted efficiently in E.coli. The predicted results of server signalP-6.0 indicated that the vaccine construct did not have a signal peptide (**Figure S2**).

#### 3.2.4 The Prediction of Immunoglobulin A-Specific B-Cell Epitopes of the Vaccine

When the screening threshold was fixed to 0.5, the IgPred server predicted that the vaccine contained 22 IgA-specific B-cell epitopes with a length of 20 amino acid residues, indicating that the vaccination was capable of inducing the secretion of IgA. The detailed results were shown in **Table S4**.

### 3.3 Secondary Structure Prediction

The secondary structure of the vaccine sequence was predicted by the Prabi server, containing 25.19% alpha-helices, 55.27% random coils, and 19.54% extended strands (**Figure S3**).

### 3.4 Three-Dimensional Modeling and Validation of the Vaccine

**Figure 3:**
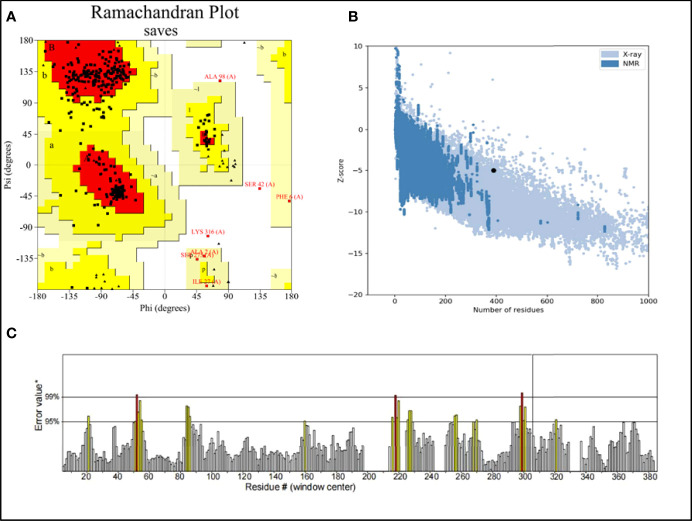
**(A)** Ramachandran plot of the vaccine. Red denotes the most favored region, dark yellow denotes the additional allowed region, light yellow denotes the generally allowed region, and white denotes the disallowed region; **(B)** three dimensional (3D) structure of the vaccine was validated by ProSA with a Z score of -5.01; **(C)** 3D structure of the vaccine was validated by ERRAT with an ERRAT score of 92.42%.

**Figure 4:**
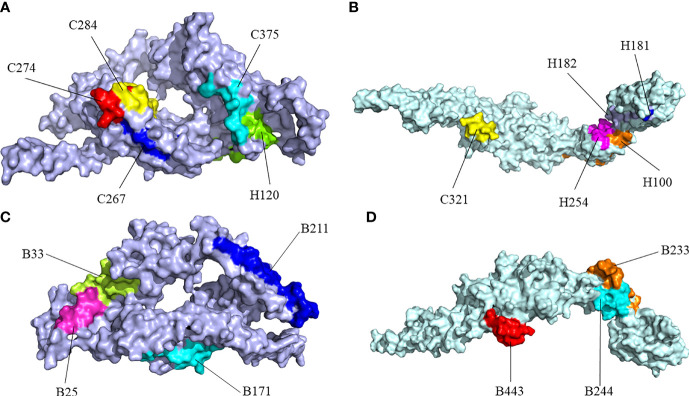
Surface positions of final selected epitopes on the three-dimensional (3D) models of the protein. **(A)** The Cytotoxicity T Lymphocytes (CTL) and Helper T lymphocyte (HTL) epitopes of CdeC. **(B)** The CTL and HTL epitopes of FliD. **(C)** The B-cell epitopes of CdeC. **(D)** The B-cell epitopes of FliD. Every CTL epitope is represented by a combination of C and its starting position. Every HTL epitope is represented by a combination of H and its starting position. Every B-cell epitope is represented by a combination of B and its starting position.

RaptorX was used to predict the primary 3D (**Figure 2B**) structures of the vaccine that were further refined *via* the GalaxyWeb server. Based on the criteria that the higher GDT-HA, the higher RMSD, and the lower MolProbity resulted in the better model, the best model was selected for further validation. The Ramachandran plot of the greatest model showed that 83% of residues situated in the most favored region, and only 0.9% lie in the disallowed region (**Figure 3A**). The Z score was -5.01 (**Figure 3B**), and the ERRAT score was 92.42 (**Figure 3C**). The Ramachandran plot, Z score, and ERRAT score of the greatest model of CdeC and FliD were shown in **Figures S4**, **S5**. The surface locations of the final antigen epitopes included in the vaccine construct were visualized on the 3D structures of the CdeC and FliD proteins (**Figure 4**).

### 3.5 Molecular Docking

**Figure 5:**
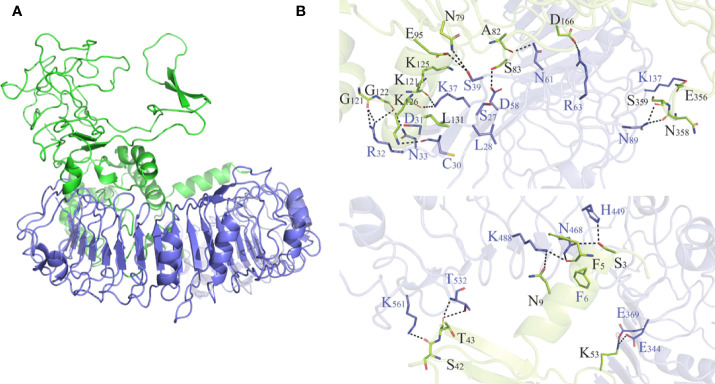
**(A)** Diagram of the lowest energy docking mode of the vaccine-Toll like receptor (TLR) 2 complex. **(B)** Pattern diagram of the binding interface of the vaccine–TLR2 complex, the TLR2 is shown in blue, the vaccine is shown in yellow-green. The binding interface amino acid of TLR2 is shown in blue, the binding interface amino acids of vaccine are shown in black. The sticks refer to binding interface amino acids, and the black dotted lines refer to hydrogen bonds.

**Figure 6:**
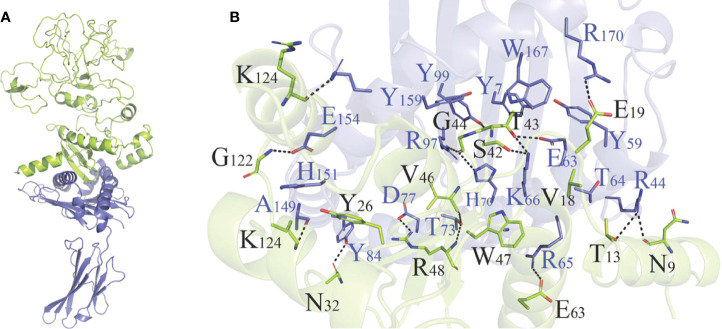
**(A)** Diagram of the lowest energy docking mode of the vaccine–HLA-A*02:01 complex. **(B)** Pattern diagram of the binding interface of the vaccine–HLA-A*02:01 complex, the HLA-A*02:01 is shown in blue, the vaccine is shown in yellow-green. The binding interface amino acid of HLA-A*02:01 is shown in blue, the binding interface amino acids of vaccine are shown in black. The sticks refer to binding interface amino acids, and the black dotted lines refer to hydrogen bonds.

**Figure 7:**
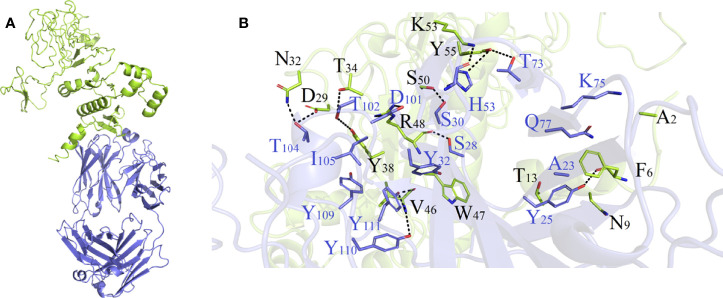
**(A)** Diagram of the lowest energy docking mode of the vaccine-B cell receptor (BCR) complex. **(B)** Pattern diagram of the binding interface of the vaccine–BCR complex, the BCR is shown in blue, the vaccine is shown in yellow-green. The binding interface amino acid of BCR is shown in blue, the binding interface amino acids of vaccine are shown in black. The sticks refer to binding interface amino acids, and the black dotted lines refer to hydrogen bonds.

As shown in **Table S5** and **Figure S6**, HADDOCK V2.4 docking data demonstrated that the vaccination had a good affinity for TLRs and MHC molecules. Also, the docking data from the clusPro server showed that there was also a good affinity between the vaccine and BCR. The surface mode of the vaccine–BCR complex docked by the clusPro server with the lowest binding energy score was shown in **Figure S7**. A total of 29 model complexes of vaccine and BCR were discovered, and the lowest binding energy score was -347.3, and the center energy was -249.6. Additionally, to reconfirm the binding mode of the vaccine with TLRs, MHC molecules, and BCR, the Rosetta was also employed. The estimated binding free energy of the best docking vaccine–TLR2 complex system was -92.0331 kcal/mol, which was divided into the hydrogen bond energy (-34.2227 kcal/mol), van der Waals energy (-21.2804 kcal/mol), and electrostatic energy (-36.5300 kcal/mol) (**Table 5**). We analyzed the binding mode of the vaccine–TLR2 complex (**Figure 5A**). The vaccine–TLR2 binding interface was shown in **Figure 5B**. Seventeen (K561, T532, K488, N468, H449, E369, E344, K126, K125, K121, E95, S83, N79, D166, E356, N358, S359) hydrophilic amino acids of the TLR2 formed intensive hydrogen-bond networks with 15 (S42, T43, N9, S3, K53, R32, N33, D31, K37, S39, D58, N61, R63, N89, K137) amino acids of the vaccine protein (**Figure 5B**), and the black dotted lines at the bonding interface referred to hydrogen bonds (**Figure 5B**). Among the polar interactions between the two proteins, there were several strong salt bridge interactions, including R63-D166, K137-E356, E369-K53, and E344-K53 (**Figure 5B**). The strength of the salt bridge is significantly stronger than that of the general hydrogen bonding, and it made a significant contribution to the stability of the protein complexes. The estimated binding free energy of the optimal vaccine–HLA-A* 02:01 complex system was -38.6189 kcal/mol, which was split into H-bond energy (-7.9353 kcal/mol), van der Waals energy (-12.3127 kcal/mol), and electrostatic energy (-18.3709 kcal/mol) (**Table 5**). The binding mode of the vaccine–HLA-A* 02:01 complex was shown in **Figure 6A**. There were 13 hydrogen bonds (K124-A149, N32-Y84, G122-E154, K124-R157, R48-T73, V46-T73, T13-R44, N9-R44, T43-E63, T43-K66, S42-K66, G44-R97, G44-H70) and 3 salt briges (R48-D77, E63-R65, E19-R170) in the binding interface of the vaccine–HLA-A*02:01 complex (**Figure 6B**). Fourteen residues have participated in the formation of hydrophobic interactions between the two proteins as shown in **Figure 6B**. Additionally, molecular docking modes between the vaccine and TLR4, TLR5, and HLA-DRB1*0401 were also performed in this study, and the binding interface diagrams of the complexes among them are shown in the supplementary material (**Figure S8**). The estimated binding free energy of the optimal vaccine–BCR complex system was -49.0037 kcal/mol, which was subdivided into H-bond energy (-12.9112 kcal/mol), van der Waals energy (-13.4489 kcal/mol), and electrostatic energy (-22.6436 kcal/mol) (**Table 5**). The binding mode of the vaccine–BCR complex was shown in **Figure 7A**. Thirteen hydrogen bonds were formed between the binding interface between the vaccine and BCR, which were the main contributing force to the binding between the two proteins (**Figure 7B**). The polar amino acids involved in the formation of hydrogen bonds include N32, D29, T34, R48, S50, K53, and Y55 in the vaccine and T104, T102, D101, S30, H53, T73, S28, K75, and Q77 in BCR (**Figure 7B**). In addition, there was good hydrophobic contact between the vaccine and BCR, such as a good hydrophobic contact between F6 and Y25 and a good hydrophobic interaction between W47 and surrounding amino acids such as Y32 and Y111 (**Figure 7B**).

**Table 5:** Docking energy of the vaccine with TLR2 and HLA-A*02:01.

Docking Statistics	Vaccine–TLR2	Vaccine–HLA-A*02:01	Vaccine–BCR
Estimated free energy of binding	-92.0331 kcal/mol	-38.6189 kcal/mol	-49.0037 kcal/mol
Hydrogen bond energy	-34.2227 kcal/mol	-7.9353 kcal/mol	-12.9112 kcal/mol
Van der Waals energy	-21.2804 kcal/mol	12.3127 kcal/mol	-13.4489 kcal/mol
Electrostatic energy	-36.5300 kcal/mol	-18.3709 kcal/mol	–22.6436 kcal/mol

TLR refers to Toll like receptors; BCR, refers to B cell receptors.

### 3.6 Molecular Dynamics Simulation

The binding free energy of vaccine–TLR2 (**Table 6**) and vaccine–HLA-*A0201 (**Table 6**) was calculated by the MMPBSA module of AMBER. In comparison to the vaccine–HLA-A*0201 complex, the vaccine–TLR2 complex was more stable, which might be explained by the fact that the larger the protein system, the more binding surfaces there are, resulting in better binding ability.

**Table 6:** Energy analysis of the vaccine–TLR2 and vaccine–HLA-A*02:01 complexes in the molecular dynamics simulation.

Energy component	Average	Std. Dev.	Std. Err. of Mean	Average	Std. Dev.	Std. Err. of Mean
	Vaccine–TLR2			Vaccine–HLA-A*0201		
VDWAALS	-152.6107	6.4819	0.9076	-164.743	9.238	1.2936
EEL	-489.7467	27.0407	3.7865	-566.1713	55.3758	7.7542
EGB	562.102	25.9777	3.6376	708.6108	50.9421	7.1333
ESURF	-21.7289	0.6819	0.0955	-24.0128	0.9258	0.1296
DELTA G gas	-642.3575	27.6305	3.869	-730.9143	54.1351	7.5804
DELTA G solv	540.3731	25.7826	3.6103	684.598	50.8602	7.1218
DELTA G binding	-101.9844	6.2414	0.874	-46.3163	8.2905	1.1609

VDWAALS, van der Waals contribution from MM; EEL, electrostatic energy as calculated by the MM force field; EGB, the electrostatic contribution to the solvation free energy calculated by Generalized Born (GB); ESURF, nonpolar contribution to the solvation free energy calculated by an empirical model; DELTA G binding, final estimated binding free energy calculated from the terms above.

**Figure 8:**
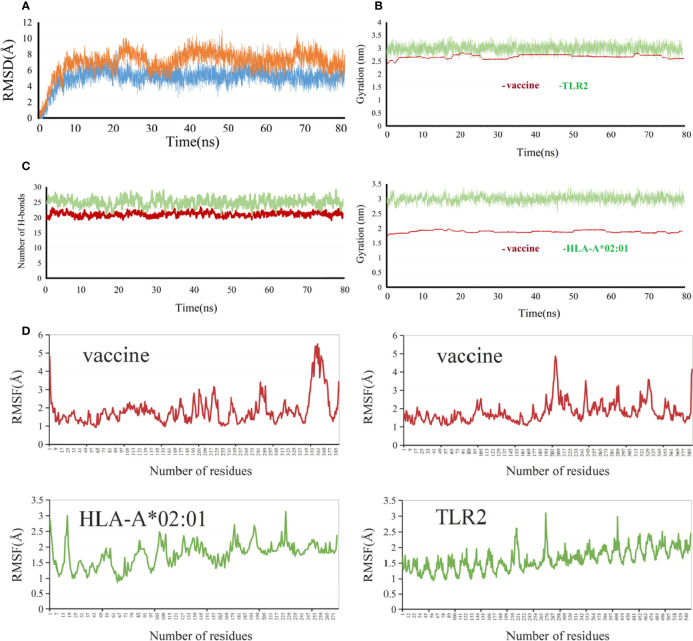
Molecular dynamics simulation analysis of the vaccine with TLR2 and HLA-A*0201. **(A)** RMSD (Root-mean-square deviation), vaccine-TLR2 (orange), vaccine-HLA-A*0201(blue) **(B)** RG (radius of gyration), **(C)** Hydrogen bonds, vaccine-TLR2 (green), vaccine-HLA-A*0201 (brown ) **(D)** RMSF (root medium square fluctuation).

The RMSD value represents the dispersion of the average value of protein centroid coordinates during the protein centroid movement, reflecting the change in the overall flexible structure of the complex system. The RMSD values (**Figure 8A**) of the vaccine–HLA-A*02:01 protein complex were relatively stable and remained almost at 5 Å within the range of 10–80 ns, indicating that the overall complex system reached a relative equilibrium state. For the vaccine–TLR2 protein complex system, the initial up and down fluctuations were relatively violent. The RMSD value (**Figure 8A**) varied most sharply within the 20–30-ns range, indicating that the complex system was extremely unstable in this interval. However, in the range of 40–80 ns, the system was stable, indicating that the protein vaccine–TLR2 structure reached a relatively balanced and stable state. The RG describes the distribution characteristics of atoms along a specific axis, elucidating the compactness of molecules. To some extent, it was capable of characterizing the overall changes in protein structure using the initial structure as a reference. In this project, the RG cyclotron radius values of vaccine–TLR2 (**Figure 8B**) and vaccine–HLA-A*02:01 protein complex systems (**Figure 8B**) were kept at a constant level, almost unchanged, indicating that the protein complexes after binding were relatively stable, which showed a similar trend to RMSD. The stability of the two complex systems was also validated by their hydrogen bonds (**Figure 8C**). The RMSF value characterizes the flexibility of amino acid residues in the protein complex. The maximum and minimum RMSF values (**Figure 8D**) of the HLA-A*02:01 molecule in the vaccine–HLA-A*02:01 complex were 3.1298 and 0.8721, respectively. RMSF showed a sustained high level in the region after 168 and was much higher in regions 192–199 and 219–228, indicating that the flexibility of the amino acid residues in these two regions was higher. The maximum and minimum RMSF values of the vaccine in the vaccine–HLA-A*02:01 complex (**Figure 8D**) were 5.4769 and 0.9279, respectively. The RMSF value of the TLR2 molecule in the vaccine–TLR2 complex was higher and fluctuated less, and its maximum and minimum RMSF values were 3.106 and 0.9486, respectively. The maximum and minimum RMSF values of the vaccine in the vaccine–TLR2 complex were 4.858 and 1.0717, respectively. The RMSF values of the vaccine showed a large fluctuation in the 201–209 region, with the maximum value reaching 4.5632.

The structures of the protein complexes of molecular docking and MD were superimposed together, and then the analysis of the changes in protein structure pre and post-MD simulation was performed (**Figure S9**). As observed, the MD simulation had a significant structural optimization effect on the protein structure complex, particularly at the protein–protein interaction interface, where the structure fluctuated significantly. The MD simulation eliminated the unfavorable factors, such as steric hindrance and binding phase difference, in the docking structure, which made the protein–protein interaction more reasonable. The post-dynamic structures were used for the following binding mode analysis, as detailed in the supplementary materials (**Figures S10, S11**). All structures in this article were visualized by Discovery Studio Visualizer 2021, UCSF Chimera1.15, and Pymol2.4.

### 3.7 Population Coverage

In this study, the multi-epitope vaccine covered 95.54% of the world’s population. In addition, in Europe and the United States, where *C. difficile* was prevalent, the population coverage also reached 96.84% and 96.86%, respectively. In China, its population coverage was relatively low, but it also reached 89.01% (**Figure S12**).

### 3.8 Immune Simulation

**Figure 9:**
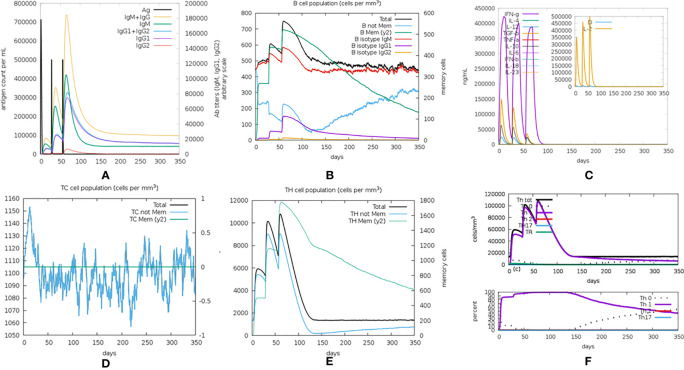
The *in silico* immune simulation results. **(A)** Immunoglobulin levels with respect to antigen concentration. **(B)** Cytokine level in response to the vaccine. **(C)** B-cell population. **(D)** TC (cytotoxic) cell population. **(E)** TH (helper) cell population. **(F)** The population of different subtypes of TH cells.

The immunogenicity of the designed vaccine was assessed through the C-ImmSim online server. Notably, the antibody titers increased significantly following the injection of the vaccine, and the increase was significantly higher after the second and third doses of injection than after the first (**Figure 9A**). After the first dose injection of the candidate vaccine, an increase in IgM antibody and the B-cell isotype IgM population and a slight elevation of B-cell isotype IgG1 population (**Figure 9B**) were detected. Following the second injection, there was a relatively large increase in B-cell isotype IgG1 and a slight increase in B-cell isotype IgG2 populations (**Figure 9B**). IgG1 + IgG2 antibodies had a higher titer than IgM antibodies in the tertiary response (**Figure 9A**). This demonstrated the Ig heavy-chain class switching, which was important for effective vaccination. Also, with the increasing immunoglobulin concentrations (i.e., IgM, IgG1, and IgG2), the concentration of antigen decreased (**Figure 9A**). Furthermore, doses of cytokines, such as IFN-γ, IL-2, and IL-12, were also seen, which showed that the proposed vaccine could cause an effective immune response (**Figure 9C**). The generation and amplification of TC (cytotoxic) cells were observed (**Figure 9D**). The increases in the TH (helper) cell population and corresponding memory cells were detected (**Figure 9E**), and TH1 was the dominant subtype (**Figure 9F**). During exposure, the macrophages (MA), natural killer (NK) cells, and dendritic cells (DCs) were also activated (**Figure S13**). Overall, these simulations demonstrated that the multi-epitope vaccine was capable of activating both cellular and humoral immunity and eliciting immune memory to induce a strong immune response to the reinvasion of the antigen.

### 3.9 *In Silico* Molecular Cloning

**Figure 10:**
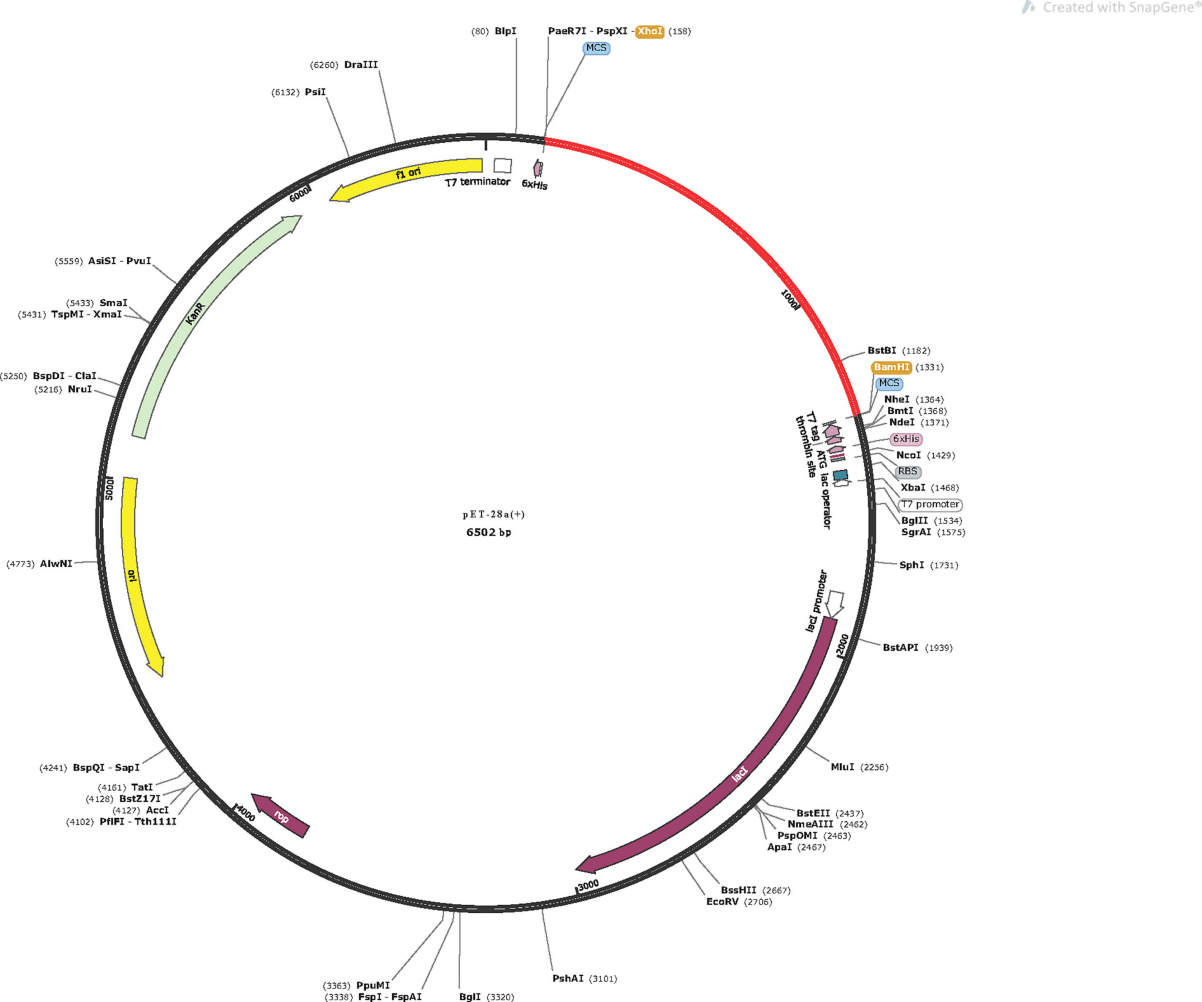
*In silico* cloning of the vaccine in the pET28a(+). Red area represents the optimized nucleotide sequence of the vaccine, the black areas represent the expression vector, pET28a(+), the XhoI and BamHI represent the cleavage sites of the restriction enzyme.

The vaccine protein was reversely translated and optimized by JCAT to acquire the codons that were highly expressed in *E. coli*. The length of the optimized codon sequences was 1,167 nucleotides with a CAI value of 1, and the GC content was 50.73%, indicating that the vaccine can be well expressed in *E. coli* and provides the possibility for the production of the vaccine (**Figure S14**). Finally, the codon sequences were successfully inserted into the pET28a(+) vector (**Figure 10**).

## 4 Discussion

CDI not only is associated with substantial morbidity and mortality worldwide but also significantly prolongs the length of hospital stay; as a result, the average cost of hospitalization greatly increases (99). Moreover, the resistance rate of *C. difficile* to its first-line treatment antibiotics is increasing, and the recurrence rate after antibiotic treatment remains at a high level (100, 101). To effectively prevent CDI, researchers have accelerated the pace of vaccine development.

Because TcdA and TcdB are the main factors associated with symptoms of CDI (102), the bulk of the earlier vaccine studies on *C. difficile* are mainly based on TcdA and TcdB. However, the failure of Sanofiofi’s vaccine during the phase 3 trial, which was once considered a potential candidate, makes it necessary to take a long, hard look at whether toxin-based vaccines work. To find another way against *C. difficile*, several surface proteins were evaluated as vaccine candidates. The SLPs have been evaluated as a vaccine component in combination with a variety of adjuvants. Different combinations induced different titers of antibodies, but no of them provided comprehensive protection for the infected model (103). Studies have shown that antibodies against several surface proteins, such as adhesin Cwp66, the protease Cwp84, the fliC and fliD, and the Fbp protein, can be observed in infected patients, which indicates that these proteins are potential vaccine candidates (35). Additionally, the protective efficacy of *C. difficile* spore proteins was also investigated (29). However, the general of these vaccination candidates failed to generate a satisfactory immunity (15, 17, 18, 29, 35). The protective efficiency of candidate vaccines designed with a single protein is limited. Therefore, can vaccines designed with multiple proteins induce a stronger protective effect?

In recent years, multi-epitope vaccines designed by an immunoinformatic method have gained increasing attention from researchers, and several multi-epitope vaccines dedicated to other pathogens have been evaluated in animal trials, and good immune effects have been achieved (22, 23). The selection of target proteins is the first and crucial step in designing a multi-epitope vaccine. It is well known that spores are the key morphological type of *C. difficile* transmission and recurrence (25). Following spore germination, adhesion and colonization in the intestinal mucosa are the initial steps for the pathogen’s settlement in the gastrointestinal tract. CdeC, a spore protein, was shown to be located in the exosporium of *C. difficile* and contributed to spore coat assembly, spore germination, and spore resistance (27, 28). According to bioinformatics analysis about the recorded genome of *C. difficile*, CdeC was highly homogeneous across *C. difficile* strains, with the lowest identity at the amino acid level exceeding 90% (104). FliD is a flagellum cap protein that has been less frequently employed in vaccine research than fliC but is more conservative (33), and it has been demonstrated to be efficient at eliciting protective immunity (35). Hence, we designed a multi-epitope vaccine combining CdeC and fliD by a bioinformatics method in this study.

In comparison to most previously studied vaccinations that only provide protective immunity to the vegetative form of *C. difficile*, this multi-epitope vaccine also produced an additional immune response against *C. difficile* spores. Moreover, to confirm that a strong immune response would be induced in the host, the designed vaccine included not only T-cell epitopes (CTL epitopes and HTL epitopes) but also B-cell epitopes. Cytokines play an important role in the host immune response (48). IFN-γ and IL-4 are two important cytokines for the activation of host immune cells; the former contributes to the differentiation of TH1 cells, while the latter plays a vital role during TH2 cell differentiation and participates in the activation of macrophage cells (45, 105). IL-2 also was a promoter of immune cell growth (50). Therefore, all selected HTL epitopes were checked for the ability to induce the generation of IFN-γ, IL-2, IL-4, and other pro-inflammatory cytokines (such as IL-1α, IL-1β, TNF-α, IL-12, IL-18, and IL-23). Based on the comprehensive consideration of the basic quantity standard and ability to induce cytokine production of the final selected HTL epitopes, only 5 HTL epitopes were included in this study. All of these 5 epitopes are IL-4-inducing and IFN-γ-inducing epitopes, and 3 of them were predicted to be inducing pro-inflammatory cytokines and IL-2. The immunity of the multi-epitope vaccine is relatively weak; the choice of adjuvant that can improve the vaccine’s immunogenicity is very important for the construction of the vaccine. HLT is a widely recognized vaccine adjuvant (106), and one of them, LT-IIb, was chosen as a vaccine adjuvant to enhance the vaccine’s immunogenicity in this study (107). The selection of linkers also is an important part for the vaccine construction, and the improper selection or inappropriate location will lead to changes in the structure of the vaccine. KK linkers were used to connect CTL epitopes and HTL epitopes, while GPGPG linkers were used to connect B-cell epitopes (108). EAAAK linker was used to connect the vaccine and adjuvant to achieve an effective connection and a fixed space between them (109, 110). We also have predicted the physicochemical properties of the designed vaccine, and its results showed that the vaccine was stable, highly soluble, and thermostable. To assess the potential of the vaccine to induce the production of IgA, we predicted the vaccine’s IgA-specific B-cell epitopes. We also performed molecular docking between the vaccine and BCR to check the interaction affinity between them, and the results showed that the vaccine could be recognized and bound by BCR. Given that antigen-activated B cells can further differentiate into sIgA-secreting plasma cells with the help of transforming growth factor-β (TGF-β) (111). Therefore, one could hypothesize that the vaccine may induce an IgA mucosal immune response. The results of molecular docking of the vaccine with TLRs and MHC molecules suggested that the designed vaccine could successfully enter the body, initiate nonspecific immunity, and subsequently initiate specific immunity. Moreover, to determine whether the vaccine construct could stably bind MHC molecules and TLRs in the free state, MD simulations were conducted. The RMSD analysis revealed that the vaccine–HLA-A*0201 complex stabilized within 10 ns, but the vaccine–TLR2 complex took longer to stabilize. The RG of the two complexes showed little fluctuation during simulation, indicating that the two complexes were stabilized during simulation, and the RMSF results demonstrated that the two complexes were both flexible. Another important part was that the immune simulation was performed in this study, and the results suggested that the designed vaccine could effectively induce a human protective immune response in theory. Additionally, population coverage suggested that the designed vaccines could reach the vast majority of the world’s population.

## 5 Conclusion

The high incidence and recurrence rate of CDI have brought great negative effects on the human economy and society. However, due to the limitations of current treatment methods and the lack of effective prevention measures, it is particularly important to develop a vaccine preventing CDI as soon as possible. In this study, a multi-epitope vaccine targeting pathogenic adhesion, sporogenesis, and spore adhesion was designed using a time-saving and low-cost approach. The antigenicity, toxicity, allergenicity, and other physicochemical properties of the vaccine were checked. The results of molecular docking and MD simulation showed that the vaccine could stably bind to TLRs and MHC molecules. Furthermore, the immune simulation suggested that the vaccine could induce a strong immune response. Finally, we evaluated the expression potential of the vaccine in *E. coli*. On all these counts, the vaccine has good efficacy against *C. difficile* in theory.

## 6 Limitations

Firstly, compared with traditional vaccines, the immunogenicity of multi-epitope vaccines is relatively weak even with the addition of adjuvants. The application of additional adjuvants such as aluminum hydroxide may resolve this problem. Secondly, CDI is an intestinal infection, but the ability of the developed vaccine to stimulate intestinal immunity remains to be further verified. Additionally, the vaccine designed in this study mainly targets the epidemic Clostridium difficile R20291, and the conserved epitopes of additional antigenic proteins will be considered in the future to improve the protective effect of the vaccine within the limited protein length. Finally, its antigenicity and immunogenicity need to be further verified in *in vivo* and *in vitro* tests.

## Data Availability Statement

All datasets presented in this study are included in the article and **Supplementary Material**.

## Author Contributions

CT, CL, and AW designed the research and analyzed the data. CT charged the drawing. CT, FZ, YX, YW, XM, SL, TL, SC, and JZ wrote the paper. CL and AW reviewed and edited the paper. CL and AW acquired the funding. All authors have read and agreed to the published version of the article.

## Funding

This research was funded by the Natural Science Foundation of Hunan Province (Award No. 2021JJ31071).

## Conflict of Interest

The authors declare that the research was conducted in the absence of any commercial or financial relationships that could be construed as a potential conflict of interest.

## Publisher’s Note

All claims expressed in this article are solely those of the authors and do not necessarily represent those of their affiliated organizations, or those of the publisher, the editors and the reviewers. Any product that may be evaluated in this article, or claim that may be made by its manufacturer, is not guaranteed or endorsed by the publisher.
