# Corrigendum: Immunoinformatics approach toward the introduction of a novel multi-epitope vaccine against *Clostridium difficile*

**DOI:** 10.3389/fimmu.2022.967126

**Published:** 2022-07-01

**Authors:** Caixia Tan, Fei Zhu, Yuanyuan Xiao, Yuqi Wu, Xiujuan Meng, Sidi Liu, Ting Liu, Siyao Chen, Juan Zhou, Chunhui Li, Anhua Wu

**Affiliations:** ^1^Infection Control Center, Xiangya Hospital, Central South University, Changsha, China; ^2^Center of Respiratory Medicine, Xiangya Hospital, Central South University, Changsha, China; ^3^National Clinical Research Center for Geriatric Disorders (XiangYa Hospital), Changsha, China

**Keywords:** *Clostridium difficile*, multi-epitope vaccine, molecular docking, molecular dynamics simulation, immunoinformatics

In the published article, there was an error. “-0.3–1” was used instead of “-1 to 1” in **section 2.7**. A correction has been made to **subsection 2.7.4 The Prediction of Immunoglobulin A-Specific B-Cell Epitopes of the Vaccine**, paragraph 1. This sentence previously stated: “The server has a filter threshold of -0.3–1, with a higher threshold implying a high probability of correct prediction but poor coverage/sensitivity”. The corrected sentence appears below:

“The server has a filter threshold of -1 to 1, with a higher threshold implying a high probability of correct prediction but poor coverage/sensitivity”.

In addition, there was an extra “the” in the final sentence of **section 2.14**. A correction has been made to paragraph 1. This sentence previously stated: “The flowchart of the the multi-epitope vaccine construction is shown in **Figure 1**”. The corrected sentence appears below:

“The flowchart of the multi-epitope vaccine construction is shown in **Figure 1**”.

In **section 3.2.4**, “20” was used instead of “22”. This sentence previously stated: “When the screening threshold was fixed to 0.5, the IgPred server predicted that the vaccine contained 20 IgA-specific B-cell epitopes with a length of 20 amino acid residues, indicating that the vaccination was capable of inducing the secretion of IgA”. The corrected sentence appears below:

“When the screening threshold was fixed to 0.5, the IgPred server predicted that the vaccine contained 22 IgA-specific B-cell epitopes with a length of 20 amino acid residues, indicating that the vaccination was capable of inducing the secretion of IgA”.

There were errors in the legend for **Table 1** as published. The “Pro-C refers to whether the HTL epitope can induce the secretion of pro-inflammatory cytokines like TNF, IL-1, IL-18, IL-12, or IL-23” should be deleted and the “Iclassalleles” should be separated into the “I class alleles”. The corrected legend appears below:

“Final selected CTL epitopes. The start position represents the starting site of the first amino acid residue of the epitope. The default minimum threshold value of the combined score was set at 0.75. Epitopes with antigenicity score >0.4 are considered to be antigenic, which can specifically bind to the antibodies or sensitized lymphocytes. Epitopes with immunogenicity score >0 are considered to be immunogenic, which can elicit an immune response. In addition, epitopes with the half-maximal inhibitory concentration (IC50) ≤500 nm and Rank% ≤2% are considered to have good binding ability to human leukocyte antigen (HLA) I class alleles. “-” refers to negative, and “+” refers to positive”.

There was an error in the legend for **Table 3** as published, “0.75” was used instead of the “0.51”. The corrected legend appears below:

“B-cell epitopes predicted using ABCpred server. The start position represents the starting site of the first amino acid residue of the epitope. The default minimum threshold value of the score was set at 0.51. Epitopes with antigenicity score>0.4 are considered to be antigenic, which can specifically bind to the antibodies or sensitized lymphocytes. “-” refers to negative”.

There were errors in the legend for **Figure 3C** as published. “PROCHECK” was used instead of “ERRAT” and “ERRAT” was used instead of “ERRAT score”. The corrected legend appears below.

**Figure 3:**
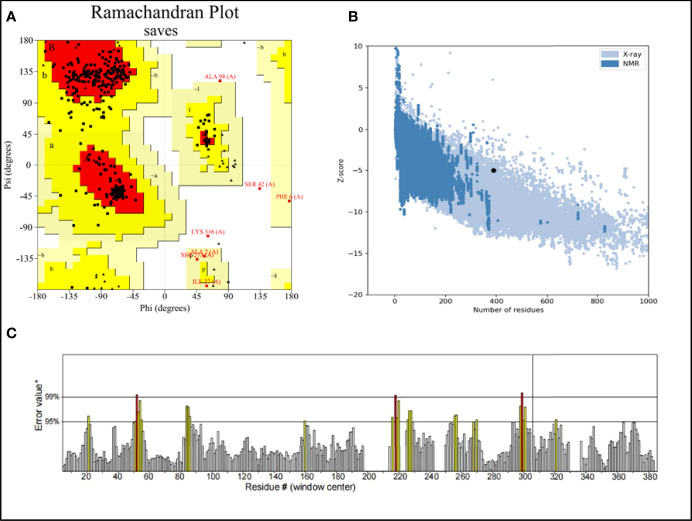
**(A)** Ramachandran plot of the vaccine. Red denotes the most favored region, dark yellow denotes the additional allowed region, light yellow denotes the generally allowed region, and white denotes the disallowed region; **(B)** three dimensional (3D) structure of the vaccine was validated by ProSA with a Z score of -5.01; **(C)** 3D structure of the vaccine was validated by ERRAT with an ERRAT score of 92.42.

There were errors in **Figure 4** and its legend as published. The amino acid residue “B33” located in **Figure 4C** was inadvertently marked in **Figure 4D** and the position of amino acid residue “B233” was inadvertently reversed from that of “B244” in **Figure 4D**. The “vaccine” in the legend for **Figure 4** should be changed into the “protein” and the “CdeC” in the legend for **Figure 4D** should be changed into the “FliD”. The corrected **Figure 4** and its legend appear below.

**Figure 4:**
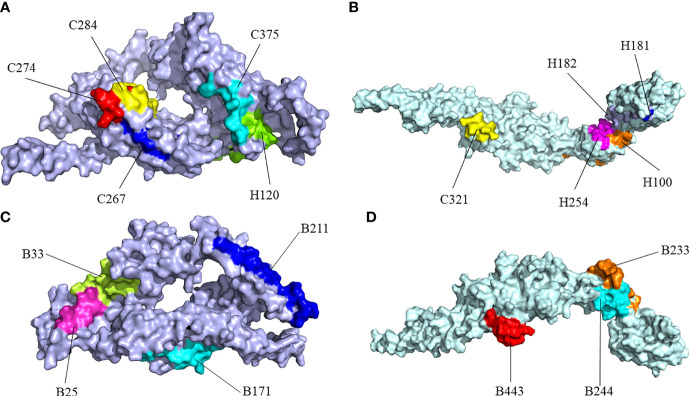
Surface positions of final selected epitopes on the three-dimensional (3D) models of the protein. **(A)** The Cytotoxicity T Lymphocytes (CTL) epitopes and helper T lymphocyte (HTL) epitopes of CdeC. **(B)** The CTL epitopes and HTL epitopes of FliD. **(C)** The B-cell epitopes of CdeC. **(D)** The B-cell epitopes of FliD. Every CTL epitope is represented by a combination of C and its starting position. Every HTL epitope is represented by a combination of H and its starting position. Every B-cell epitope is represented by a combination of B and its starting position.

There there were errors in **Figure 10** and its legend as published. Chinese text was used, instead of English, and “ Red “ was used instead of the “ Red areas “ in the legend. The corrected **Figure 10** and its legend appear below.

**Figure 10:**
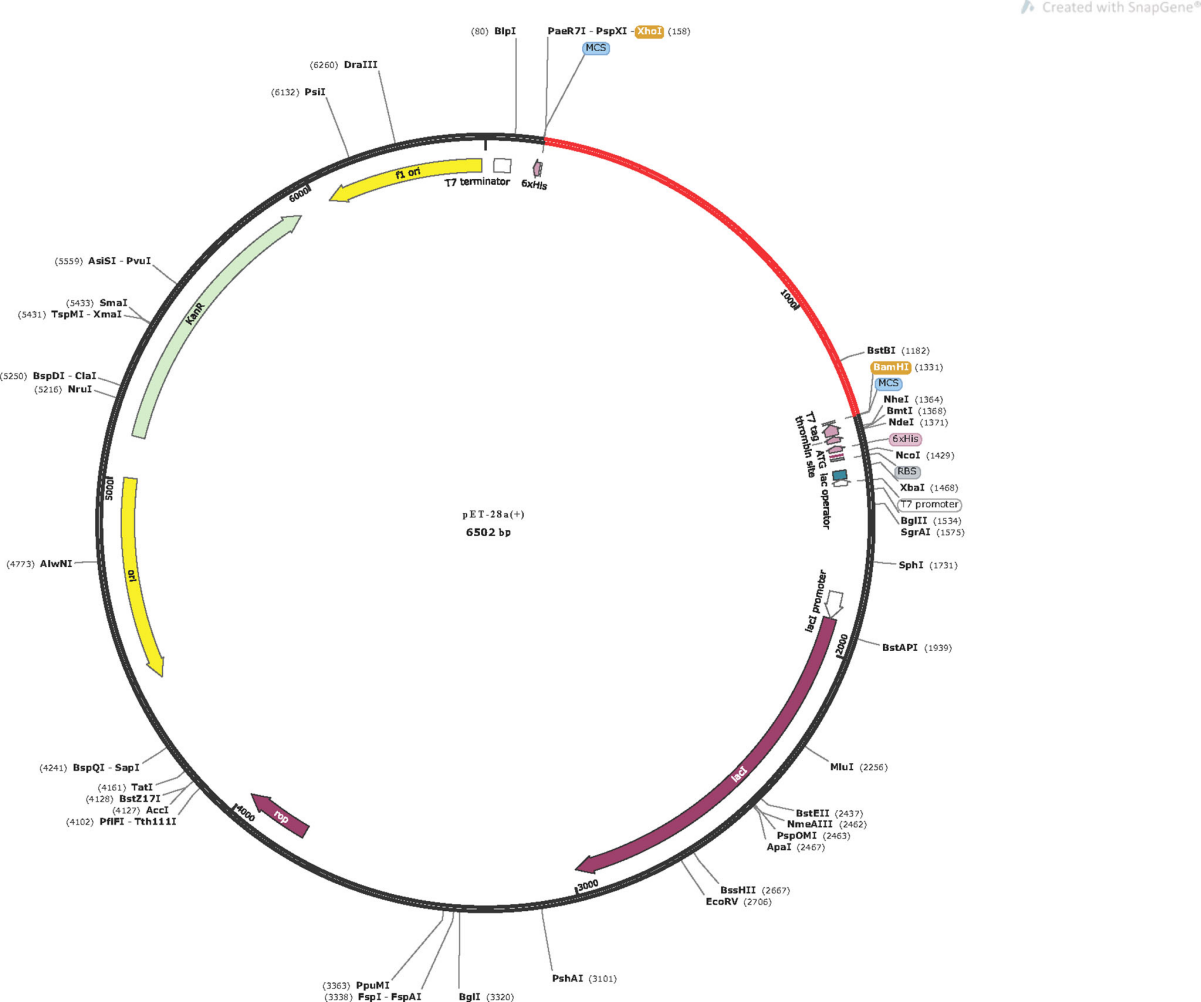
In silico cloning of the vaccine in the pET28a(+). Red areas represent the optimized nucleotide sequence of the vaccine, the black areas represent the expression vector, pET28a(+), the XhoI and BamHI represent the cleavage sites of the restriction enzyme.

There were errors in the legends for **Supplementary Figure S4C**, **Figure S5A** and **Figure S5C**. “PROCHECK” was used instead of “ERRAT” and “ERRAT” was used instead of “ERRAT score” in the legends of **Figure S4C** and **Figure S5C**. In addition “CdeC” was used instead of “FliD” in the legend of **Figure S5A**. The corrected legends for **Figures S5C**, **S5A**, and **S5C** appear below:

Figure S4 | (C) 3D structure of the vaccine was validated by ERRAT with an ERRAT score of 80.47.

Figure S5 | (A) Ramachandran Plot of the FliD: Red denotes the most favoured region (90.1%), dark yellow denotes the additional allowed region (9%), light yellow denotes the generally allowed region, and white denotes the disallowed region (0.9%). (C) 3D structure of vaccine was validated by “ERRAT” with an “ERRAT score” of 86.81.

There were errors in Supplementary (The horizontal header of **Table S1**, **Table S2**, and **Table S3**). “Aimilarity“ was used instead of “similarity“ in the horizontal header of **Table S1**, **Table S2** and **Table S3**, and the position of “Epitope” was inadvertently switched with that of “Start position” and “Combined core” was used instead of “Score”. in the horizontal header of **Table S3**. The correct horizontal headers of **Table S1**, **Table S2** and **Table S3** appear below.

Finally, there were errors in **Supplementary Figure S10**. The images in **Figure S10** were out of order. The correct **Figure S10** appears below:

Figure S10 | Surface diagram of protein electrostatic interaction in molecular dynamic simulation. (A) Vaccine (B) Receptors (C) Vaccine (Rotate 90°) (D) Receptors (Rotate 90°). (a) Vaccine-TLR2 (b)Vaccine-HLA-A*0201.

The authors apologize for these errors and state that this does not change the scientific conclusions of the article in any way. The original article has been updated.

## Publisher’s note

All claims expressed in this article are solely those of the authors and do not necessarily represent those of their affiliated organizations, or those of the publisher, the editors and the reviewers. Any product that may be evaluated in this article, or claim that may be made by its manufacturer, is not guaranteed or endorsed by the publisher.

