# Supplementary material for: Immunoinformatics Approach Toward the Introduction of a Novel Multi-Epitope Vaccine Against Clostridium difficile
Source: Front Immunol. 2022 May 26;13:887061. doi: 10.3389/fimmu.2022.887061 (PMC9204425; doi:10.3389/fimmu.2022.887061)
Supplement: Supplementary file 1 [file DataSheet_1.docx]

**
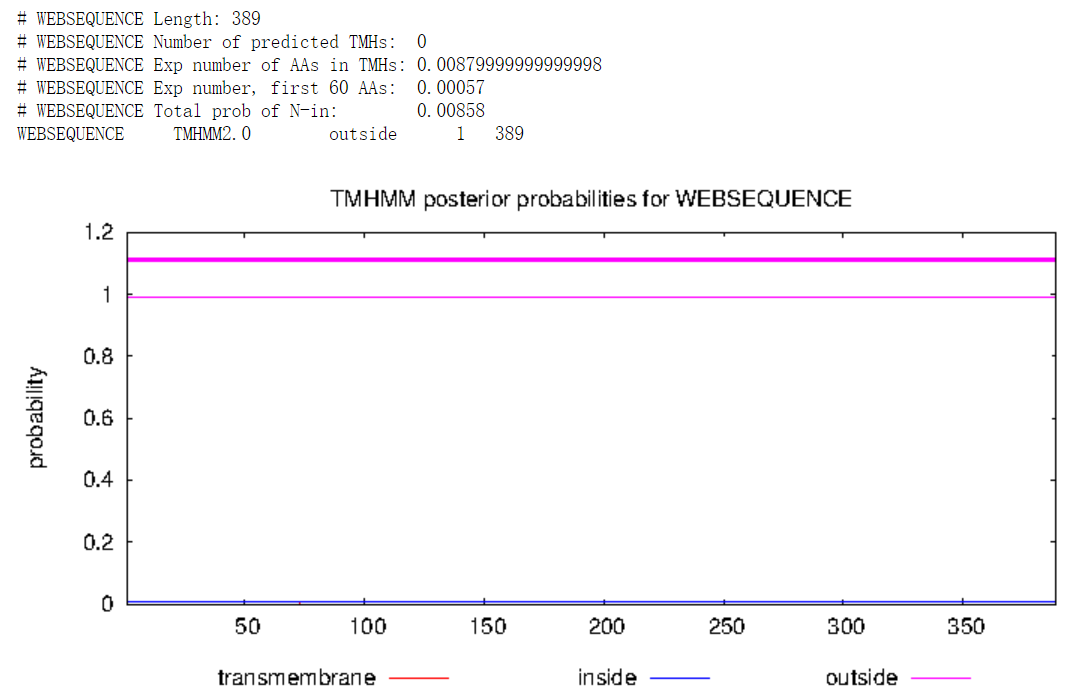
**

**Figure S1.**The transmembrane helix of designed vaccine


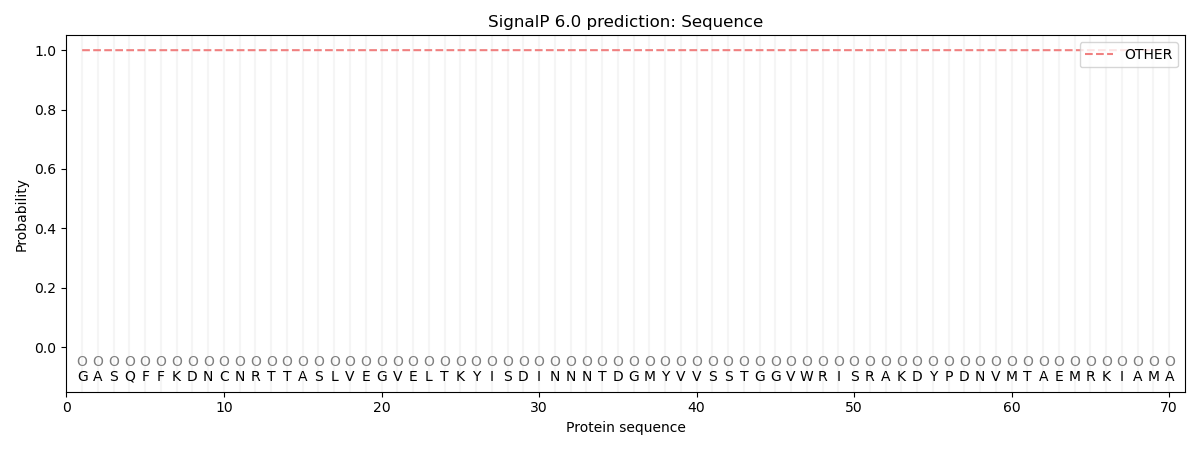


**Figure S2.**The predicted signal peptide of designed vaccine


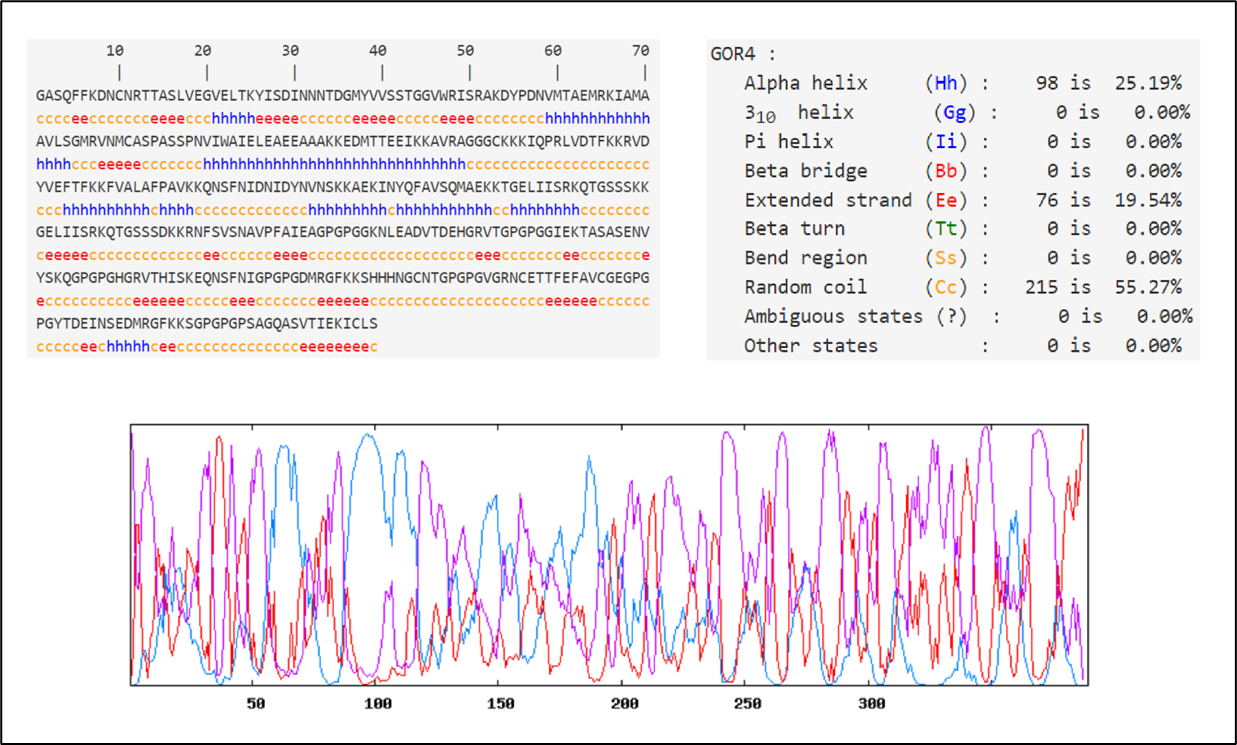


**Figure S3.** The secondary structure of the vaccine sequence.


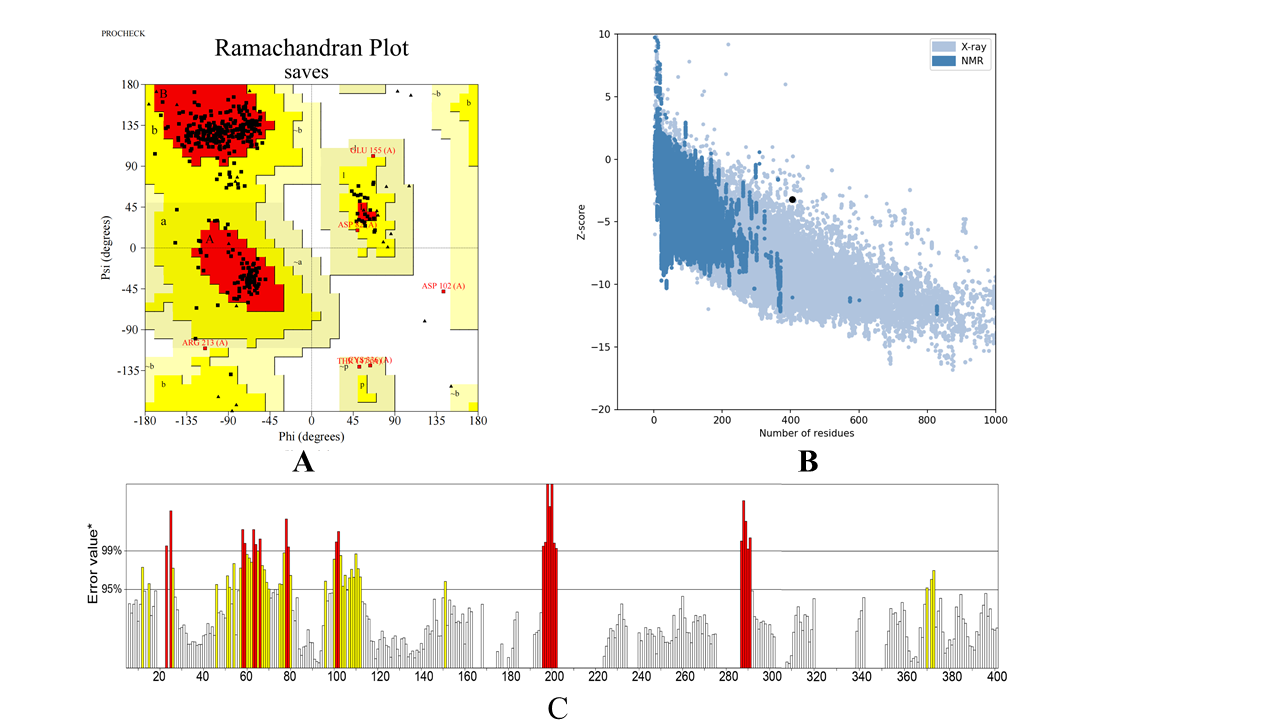
**Figure S4**. (A) Ramachandran Plot of the CdeC: Red denotes the most favoured region(84.3%), dark yellow denotes the additional allowed region(15.3%), light yellow denotes the generally allowed region, and white denotes the disallowed region(0.4%). (B) 3D structure of vaccine was validated by ProSA with a Z-score of -3.2; (C) 3D structure of vaccine was validated by ERRAT with an ERRAT score of 80.47.

**
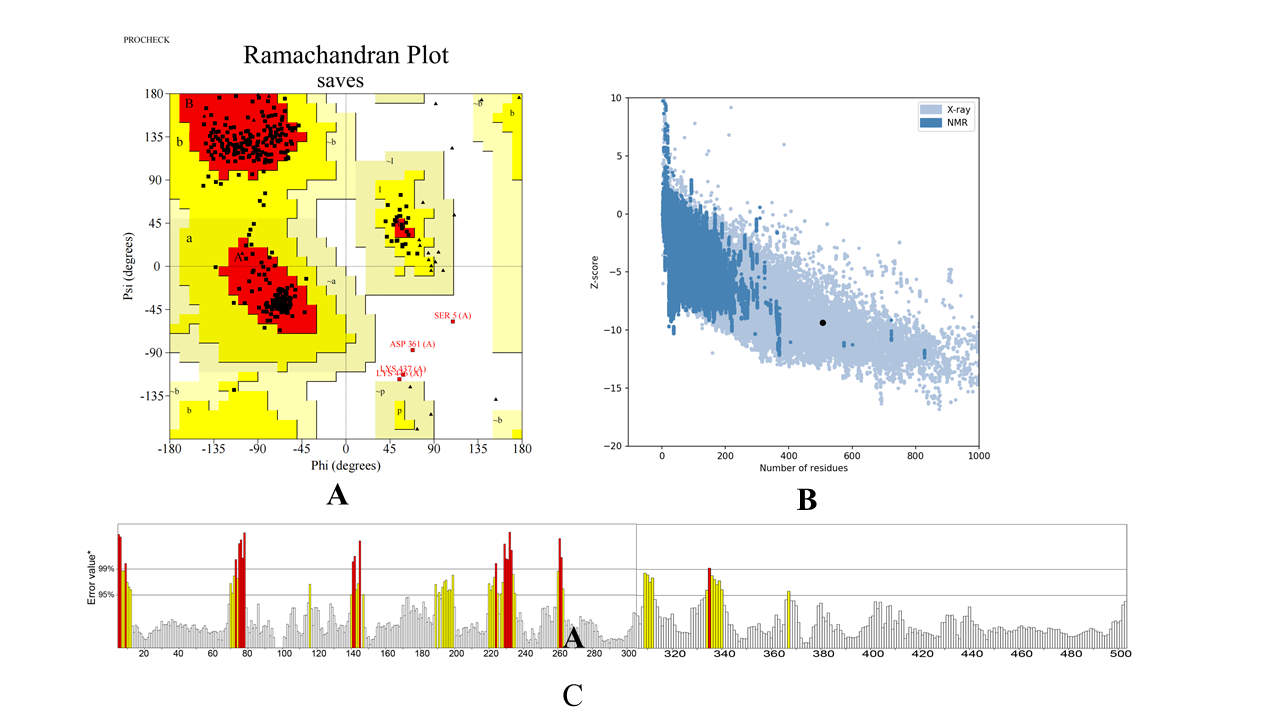
Figure S5**. (A) Ramachandran Plot of the FliD: Red denotes the most favoured region(90.1%), dark yellow denotes the additional allowed region(9%), light yellow denotes the generally allowed region, and white denotes the disallowed region(0.9%). (B) 3D structure of vaccine was validated by ProSA with a Z-score of -9.38. (C) 3D structure of vaccine was validated by ERRAT with an ERRAT score of 86.81.


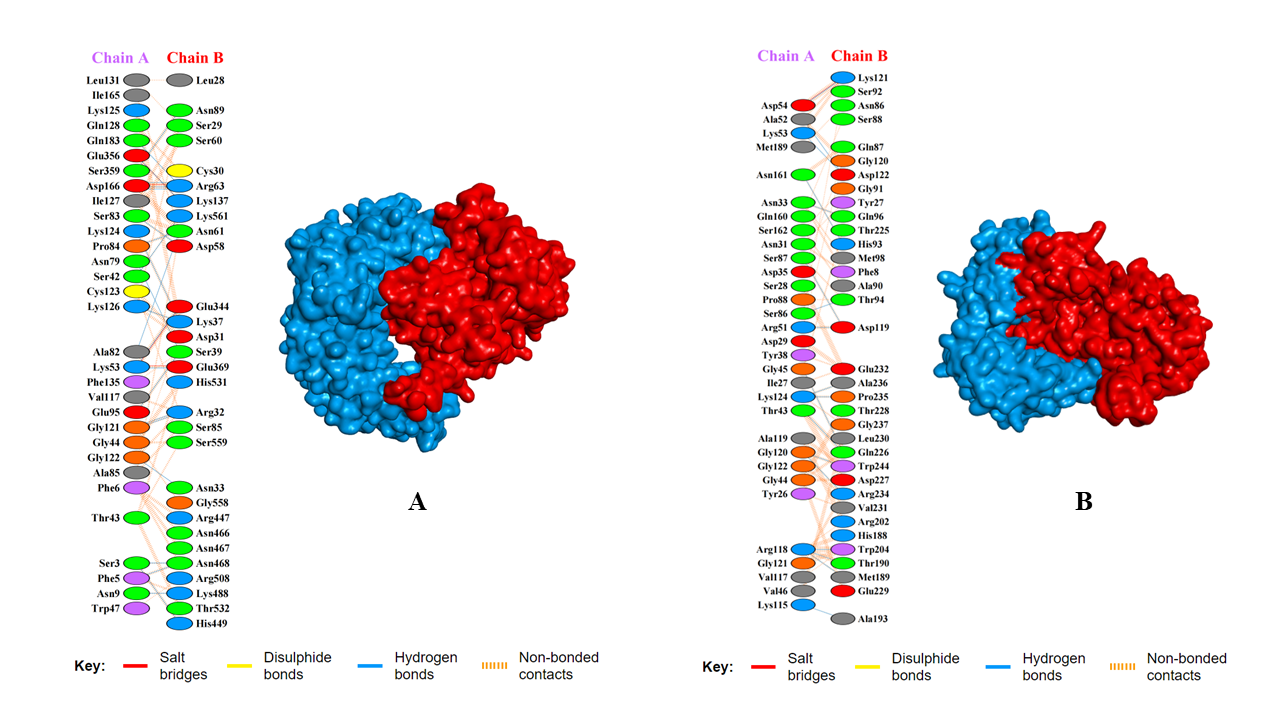


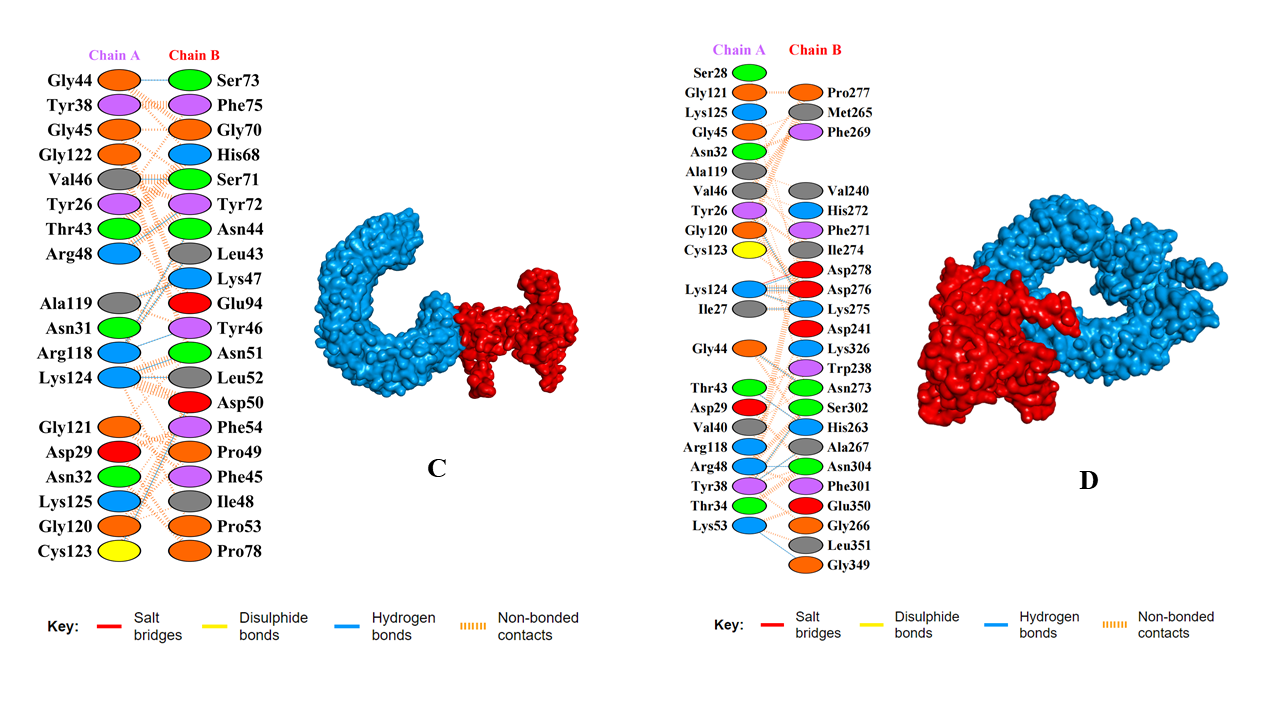


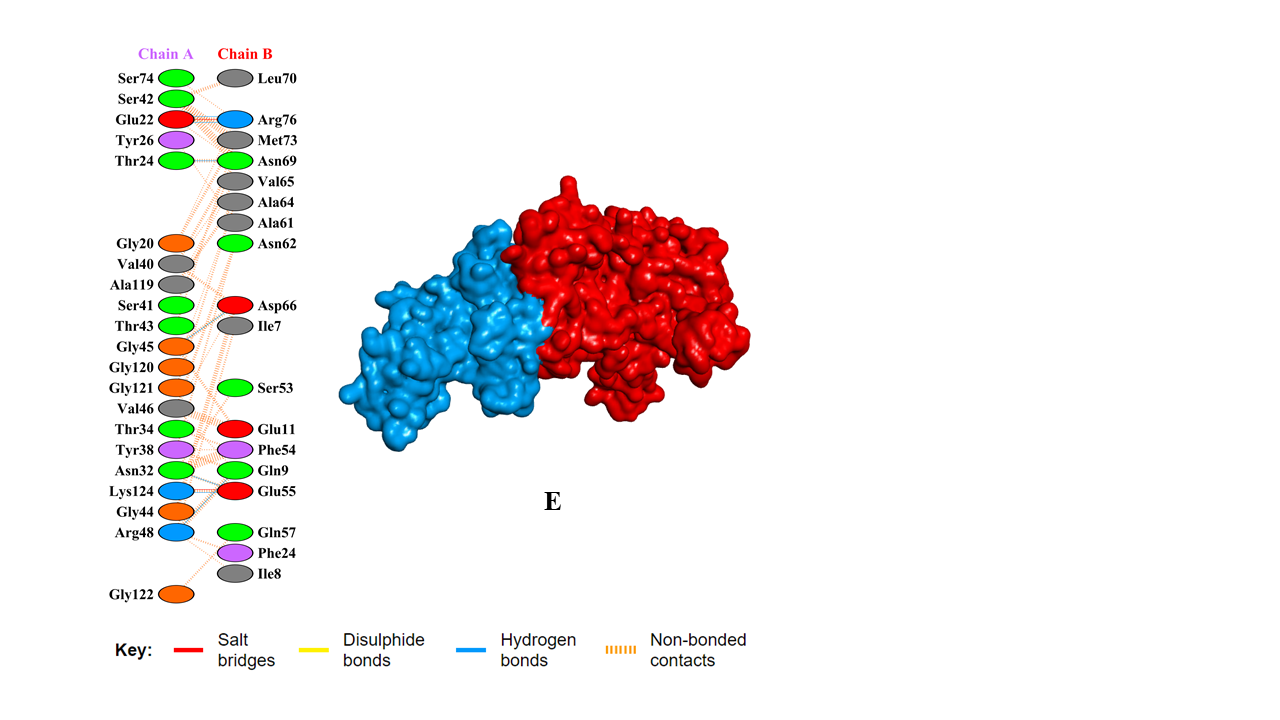
**Figure S6**. Docked complex of designed vaccine(red) and receptors (blue) and residue interactions across the protein-protein interface; (A) Vaccine-TLR2 complex, (B) Vaccine-HLA-A*0201 complex, (C) Vaccine-TLR4 complex, (D) Vaccine-TLR5 complex, (E) Vaccine- HLA-DRB1*0401 complex.

**
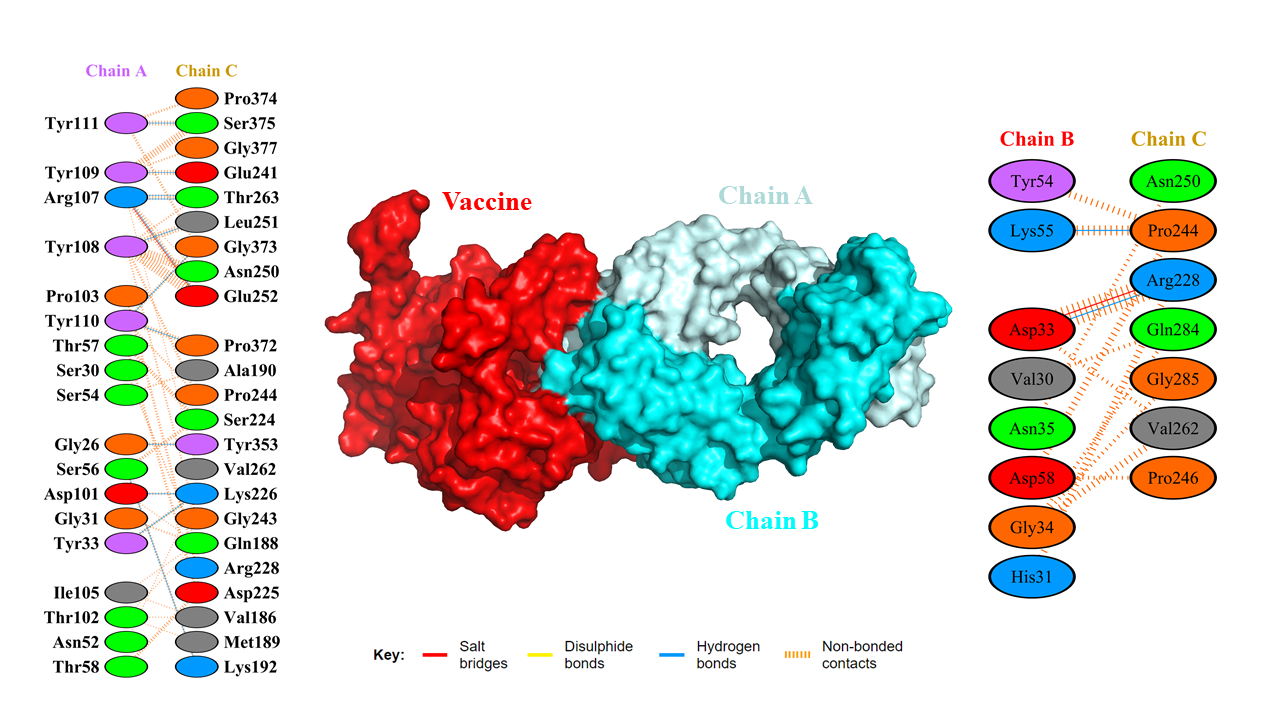
Figure S7.** The surface mode of vaccine-BCR docked complex and residue interactions across the protein-protein interface


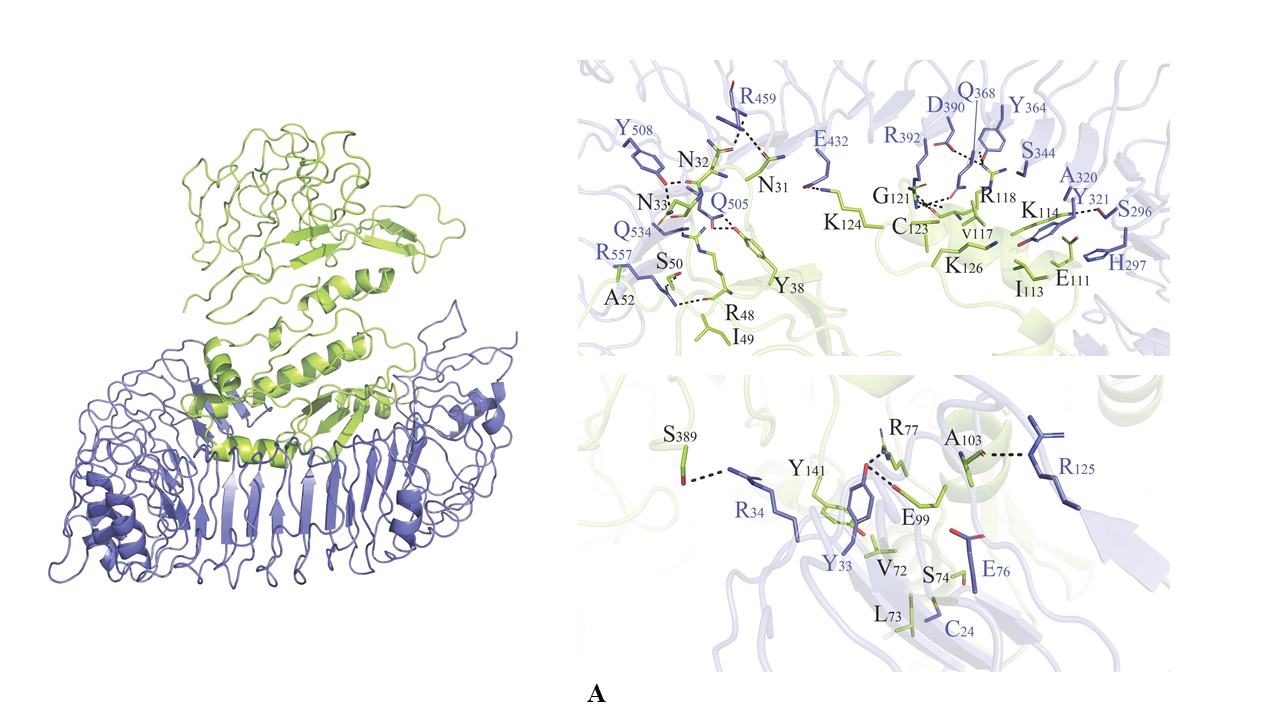


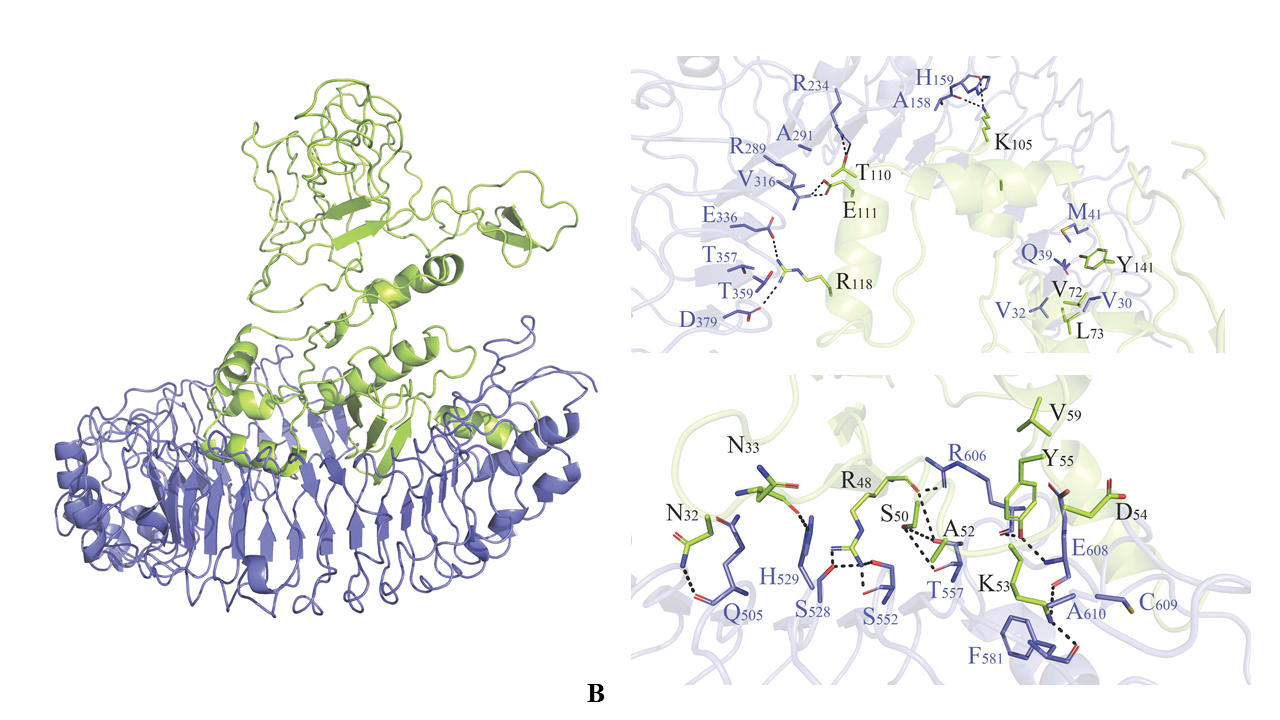


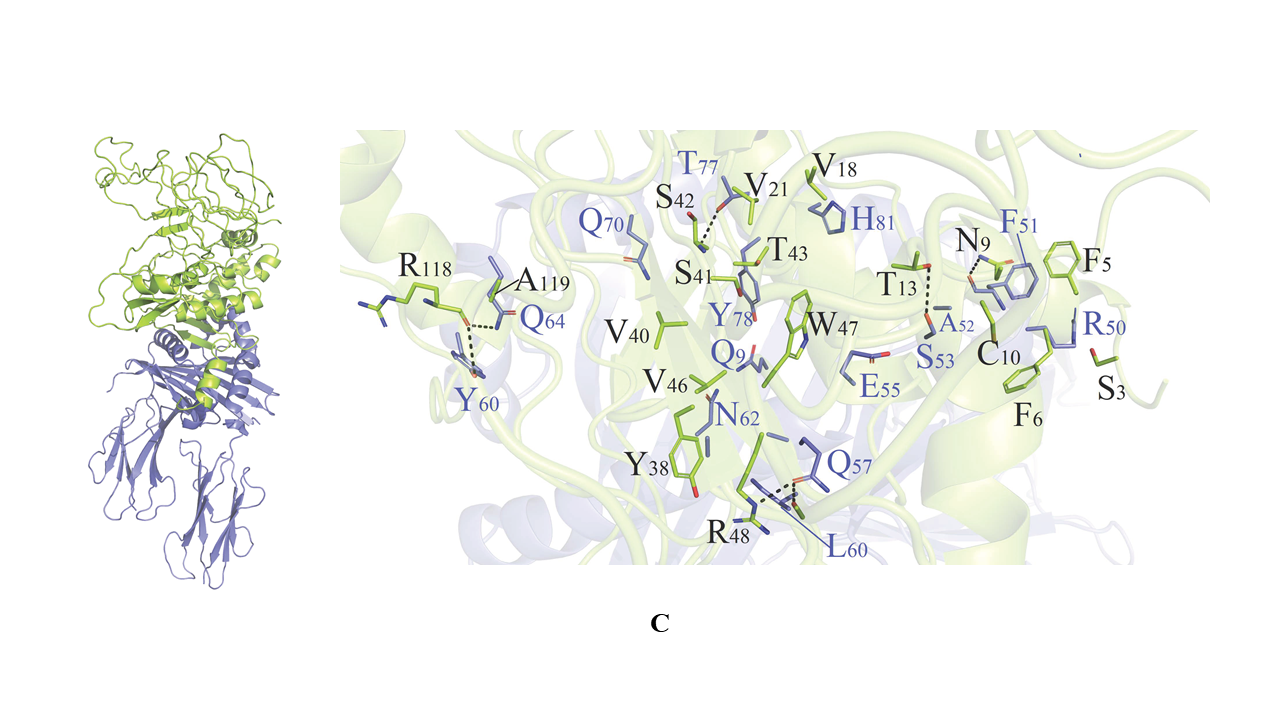
**Figure S8.** The diagram of the lowest energy docking mode and the pattern diagram of the binding interface of the vaccine-receptors complex; the receptors showing with blue, the vaccine showing with yellow-green; The binding interface amino acid of receptors showing with blue; the binding interface amino acids of vaccine showing with black. The sticks refer to binding interface amino acids, and the black dotted lines refer to hydrogen bonds. (A) Vaccine-TLR5 complex (B) Vaccine-TLR4 complex (C) Vaccine -HLA-DRB1*0401 complex.

**
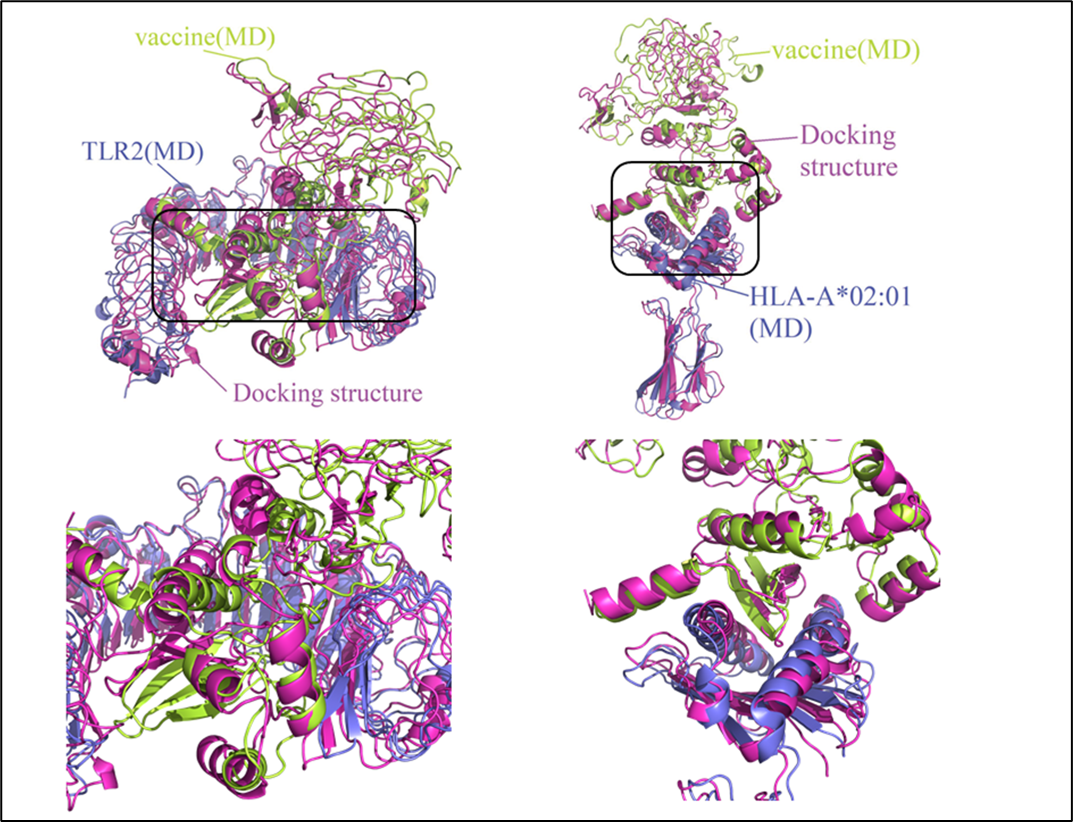
Figure S9.** The structural changes between docking complex and dynamic simulation complex.

**
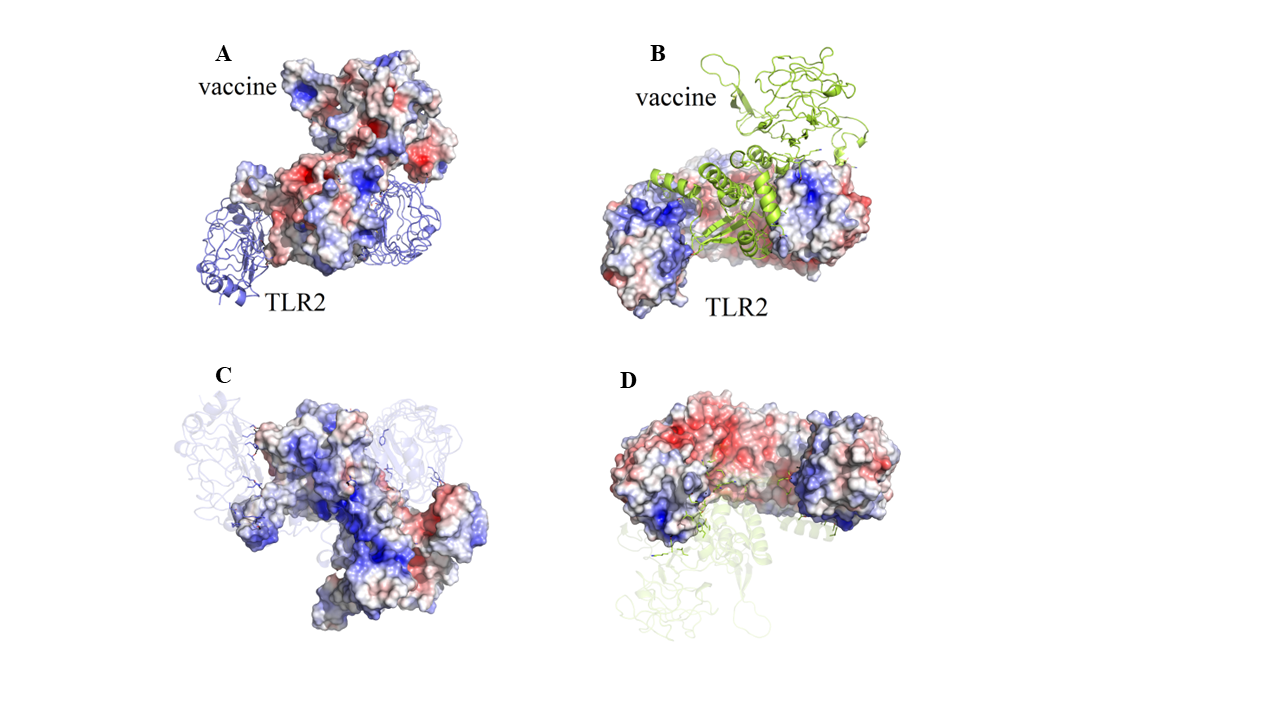
**

**（a）**


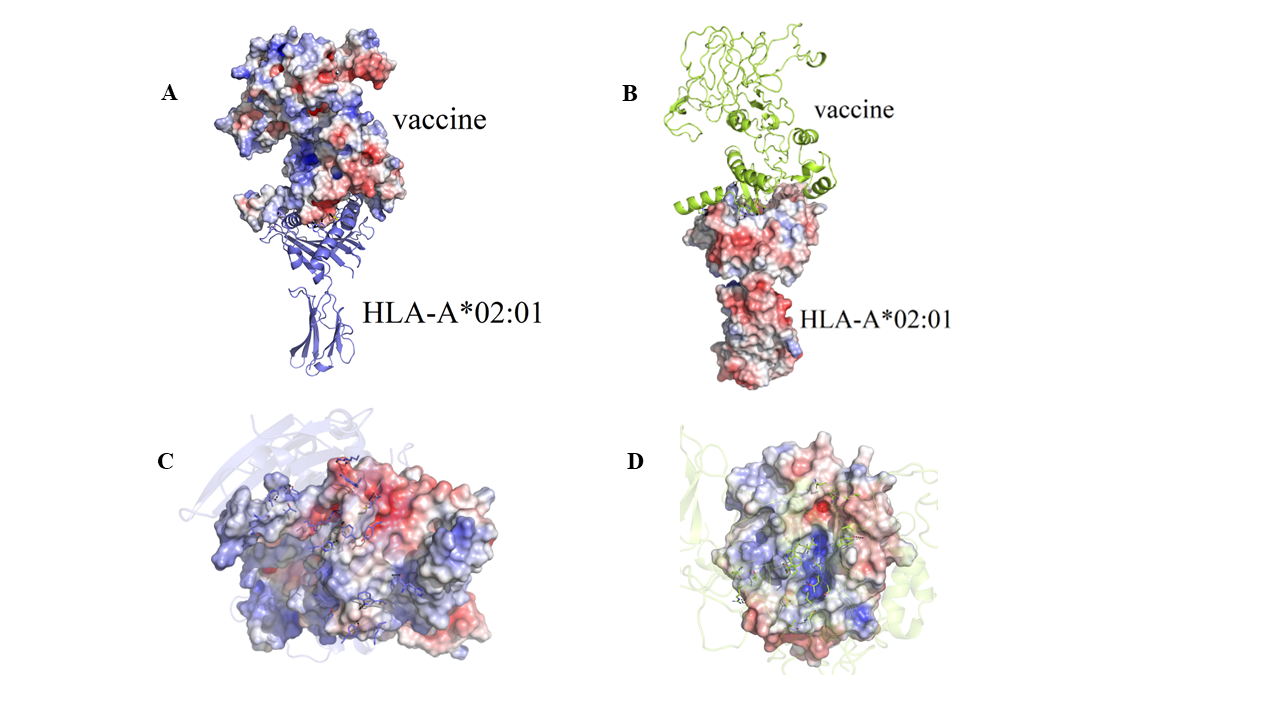


**（b）**

**Figure S10.** Surface diagram of protein electrostatic interaction in molecular dynamic simulation. (A) Vaccine (B) Receptors (C) Vaccine (Rotate 90 °) (D) Receptors (Rotate 90 °). (a) Vaccine-TLR2 (b)Vaccine-HLA-A*0201.


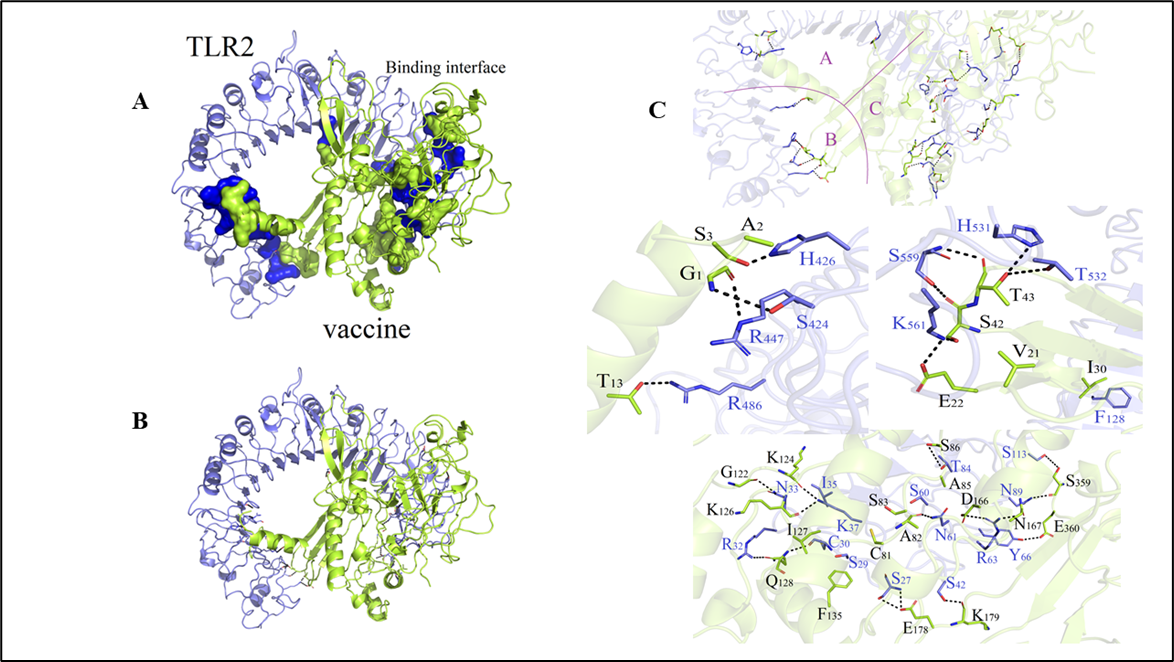


**(a)**

**
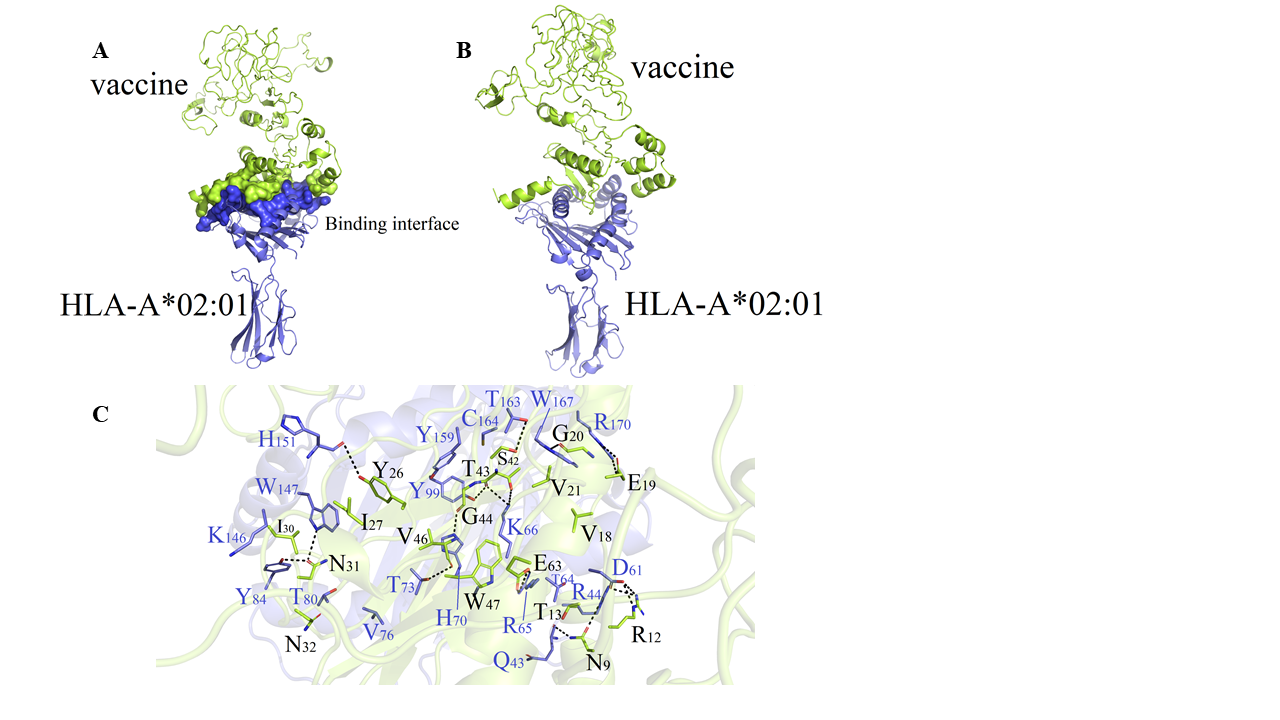
（b）**

**Figure S11.** The analysis of protein interaction patterns in molecular dynamic simulation (A) Integrated binding mode diagram (B) Binding mode diagram (C) Detailed binding mode diagram. The receptors showing with blue, the vaccine showing with yellow-green; The binding interface amino acid of receptors showing with blue, and the binding interface amino acids of the vaccine showing with black. The sticks refer to binding interface amino acids, and the black dotted lines refer to hydrogen bonds. (a) Vaccine-TLR2, (b)Vaccine-HLA-A*0201.

**
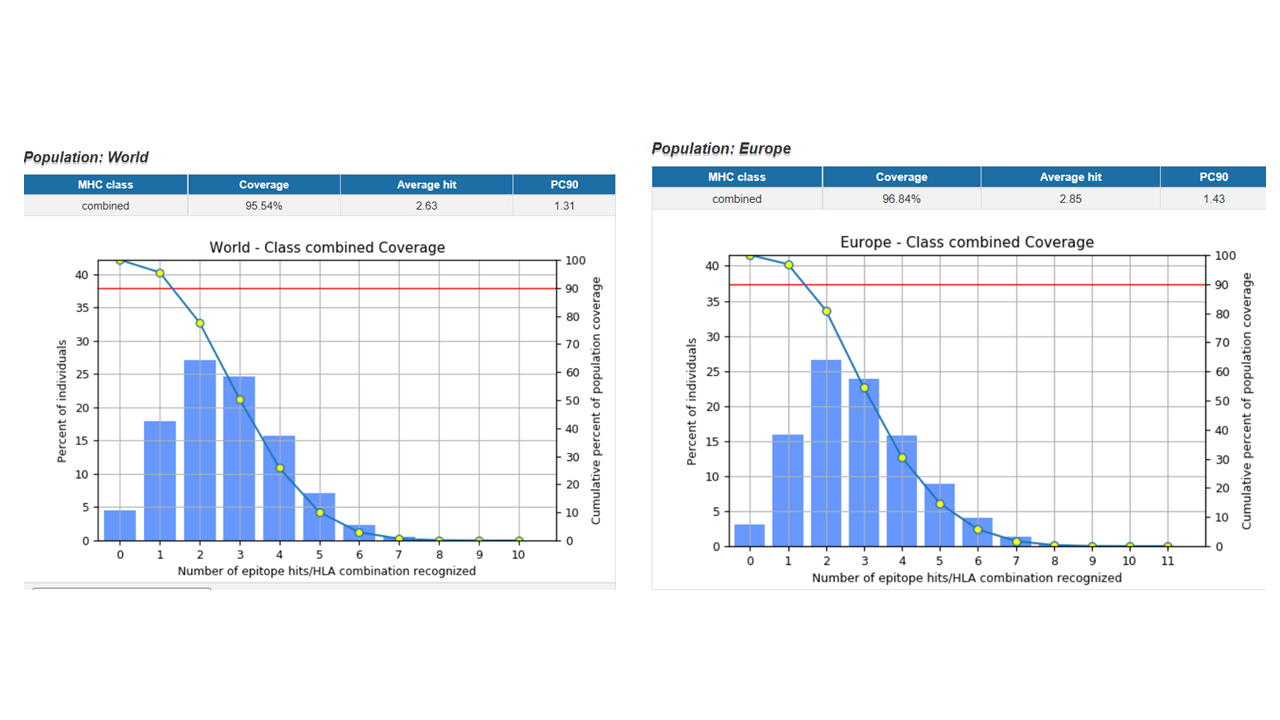
(a)**


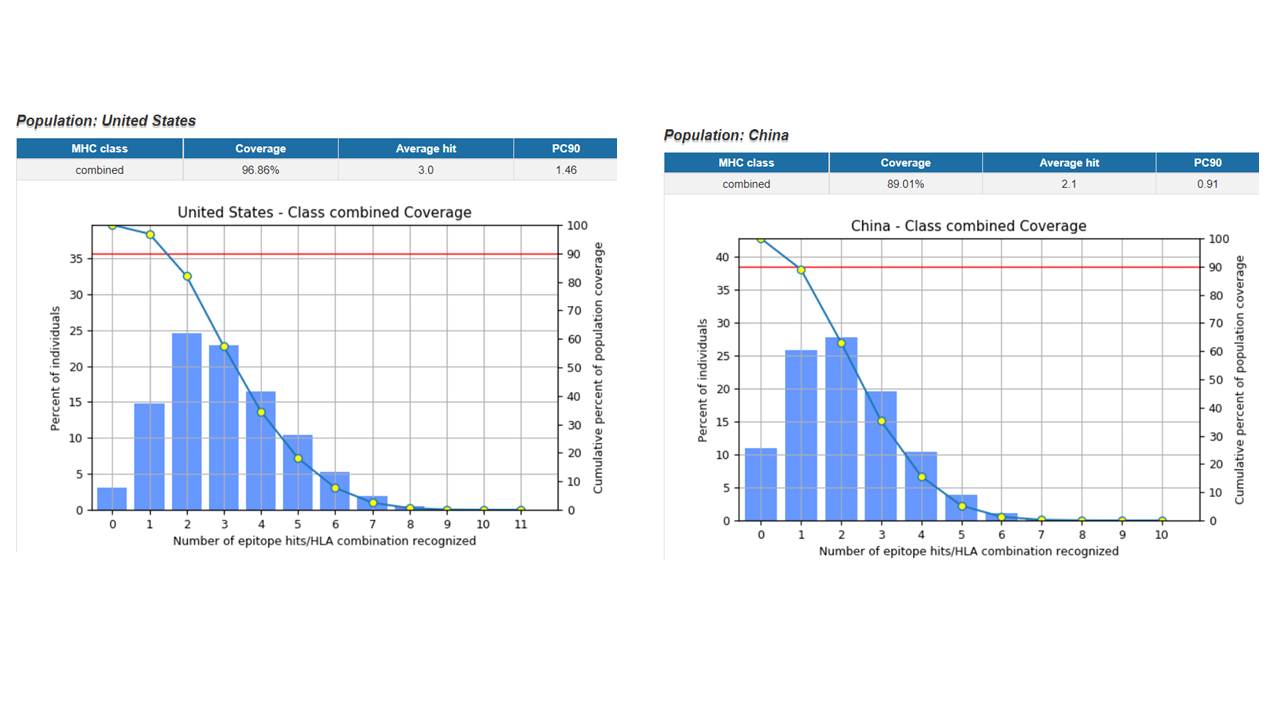


**(b)**

**Figure S12.** Population coverage of different areas.

**Figure S13.** The immune simulation results of the vaccine candidate. (A ) macrophages (MA) cells levels , (B) Natural killer (NK) cells levels, (C) dendritic (DC) cells levels, (D)epithelial (EP) cell
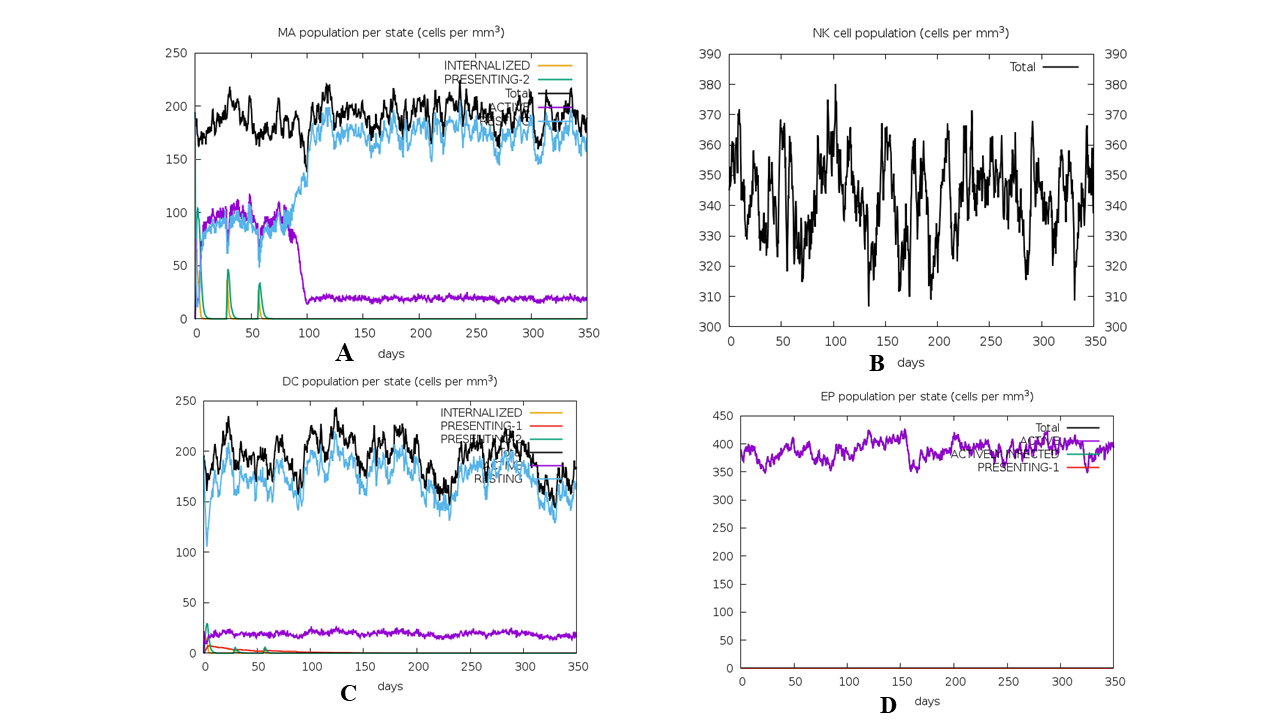
s levels.


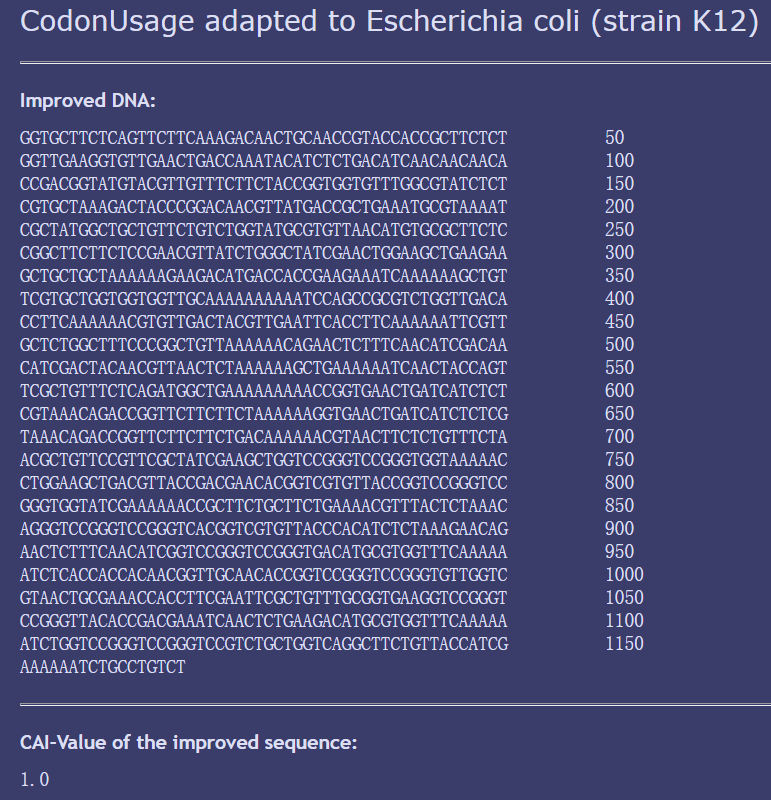


**Figure S14.** The optimized nucleotide sequences of the vaccine.
